# Supplementary material for: A parabrachial-hypothalamic parallel circuit governs cold defense in mice
Source: Nat Commun. 2023 Aug 15;14:4924. doi: 10.1038/s41467-023-40504-6 (PMC10427655; doi:10.1038/s41467-023-40504-6)
Supplement: Supplementary file 1 — Supplementary Information [file 41467_2023_40504_MOESM1_ESM.pdf]

Supplementary Fig. 1 | Projections of LPB glutamatergic neurons throughout the brain.

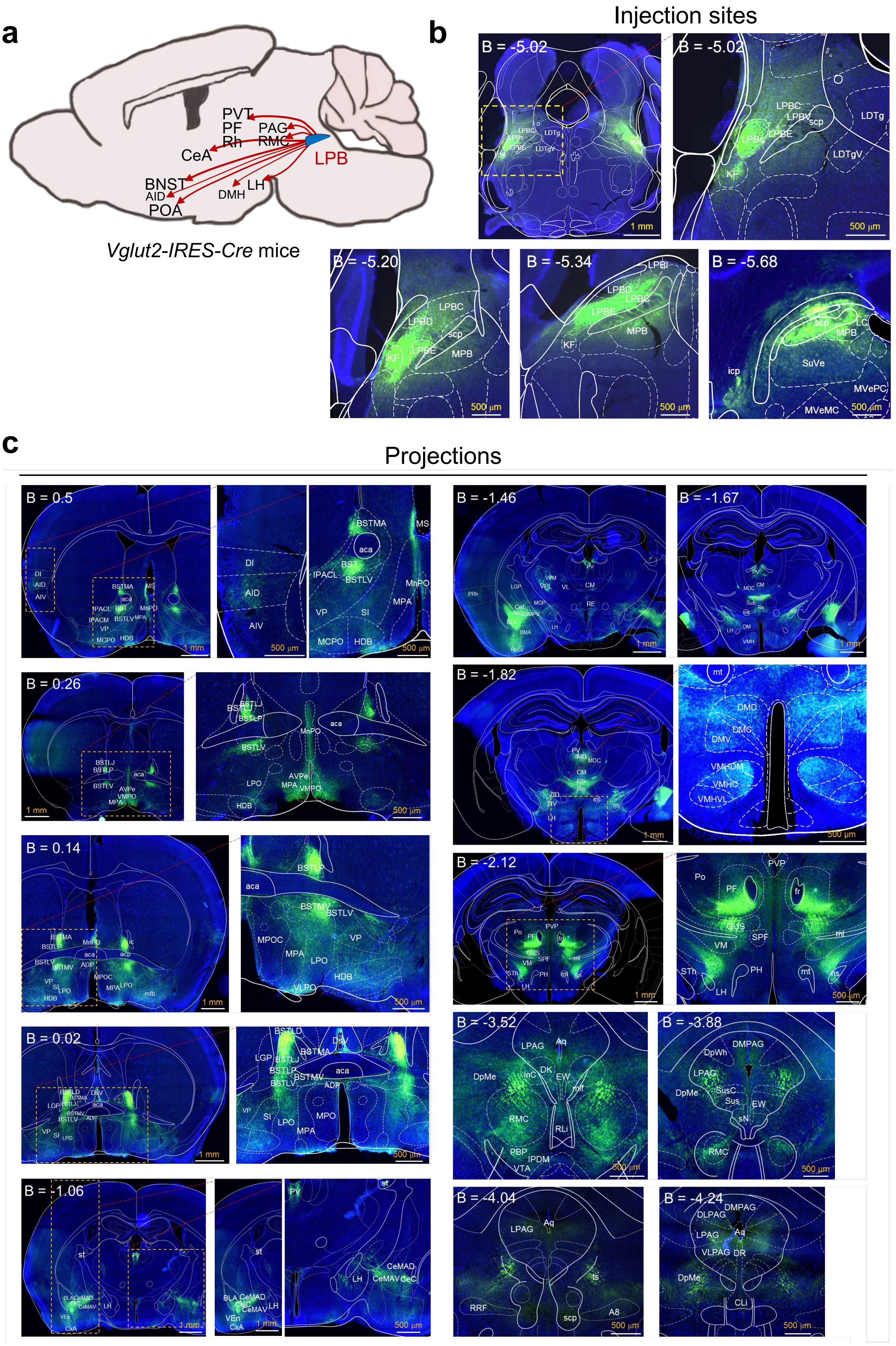

Supplementary Fig. 1 | Projections of LPB glutamatergic neurons throughout the brain.

(a) The axonal projection pattern of LPB glutamatergic neurons throughout the brain, revealed by anterograde tracing AAVs. Injection sites of AAV9-DIO-ChR2-eYFP in the LPB are indicated in the figure. Thicker lines indicate stronger eYFP expression.

(b) Representative images are showing ChR2-eYFP expression in the injection sites. This experiment was repeated at least 10 times independently with similar results.

(c) Representative images are showing ChR2-eYFP expression in axonal terminals at various brain sites. This experiment was repeated at least 10 times independently with similar results.

B, bregma; LPBC, lateral parabrachial nucleus, central part; LPBD, lateral parabrachial nucleus, dorsal part; LPBE, lateral parabrachial nucleus, external part; LPBV, lateral parabrachial nucleus, ventral part; LPBI, lateral parabrachial nucleus, internal part; LDTg, laterodorsal tegmental nucleus; LDTgV, laterodorsal tegmental nucleus, ventral part; KF, Kolliker-Fuse nucleus; MPB, medial parabrachial nucleus; LC, locus coeruleus; MVeMC, medial vestibular nucleus, magnocellular part; MVePC, medial vestibular nucleus, parvicellular part; icp, inferior cerebellar peduncle; scp, superior cerebellar peduncle; SuVe, superior vestibular nucleus. PAG, periaqueductal gray matter; SC, superior colliculus; RVM, rostral ventromedial medulla; PnO, the oral part of pontine reticular nucleus; TH, thalamus; Po, posterior thalamic nuclear group; POA, preoptic nucleus; PaF, parafascicular thalamic nucleus; SPF, subparafascicular thalamic nucleus; VPM, ventral posteromedial thalamic; ZID, zona incerta, dorsal part; DLPAG, dorsolateral periaqueductal gray; DMPAG, dorsomedial periaqueductal gray; LPAG, lateral periaqueductal gray; InG, intermediate gray layer of the superior colliculus; InWh, intermediate white layer of the superior colliculus; DpG, deep gray layer of the superior colliculus; Gi, gigantocellular reticular nucleus; GiA, gigantocellular reticular nucleus, alpha part; LPGi, lateral paragigantocellular nucleus; rmg, raphe magnus nucleus; LPO, lateral preoptic area; StHY, striohypothalamic nucleus; MPA, medial preoptic area; MPOM, medial preoptic nucleus, medial part; SIB, substantia innominata, basal part; HDB, nucleus of the horizontal limb of the diagonal band; D3V, dorsal 3rd ventricle; PVT, paraventricular thalamic nucleus.

Supplementary Fig. 2 | Mapping the collateral projections of LPB^{Vglut2} neurons to the POA (MnPO & VMPO) and the DMH.

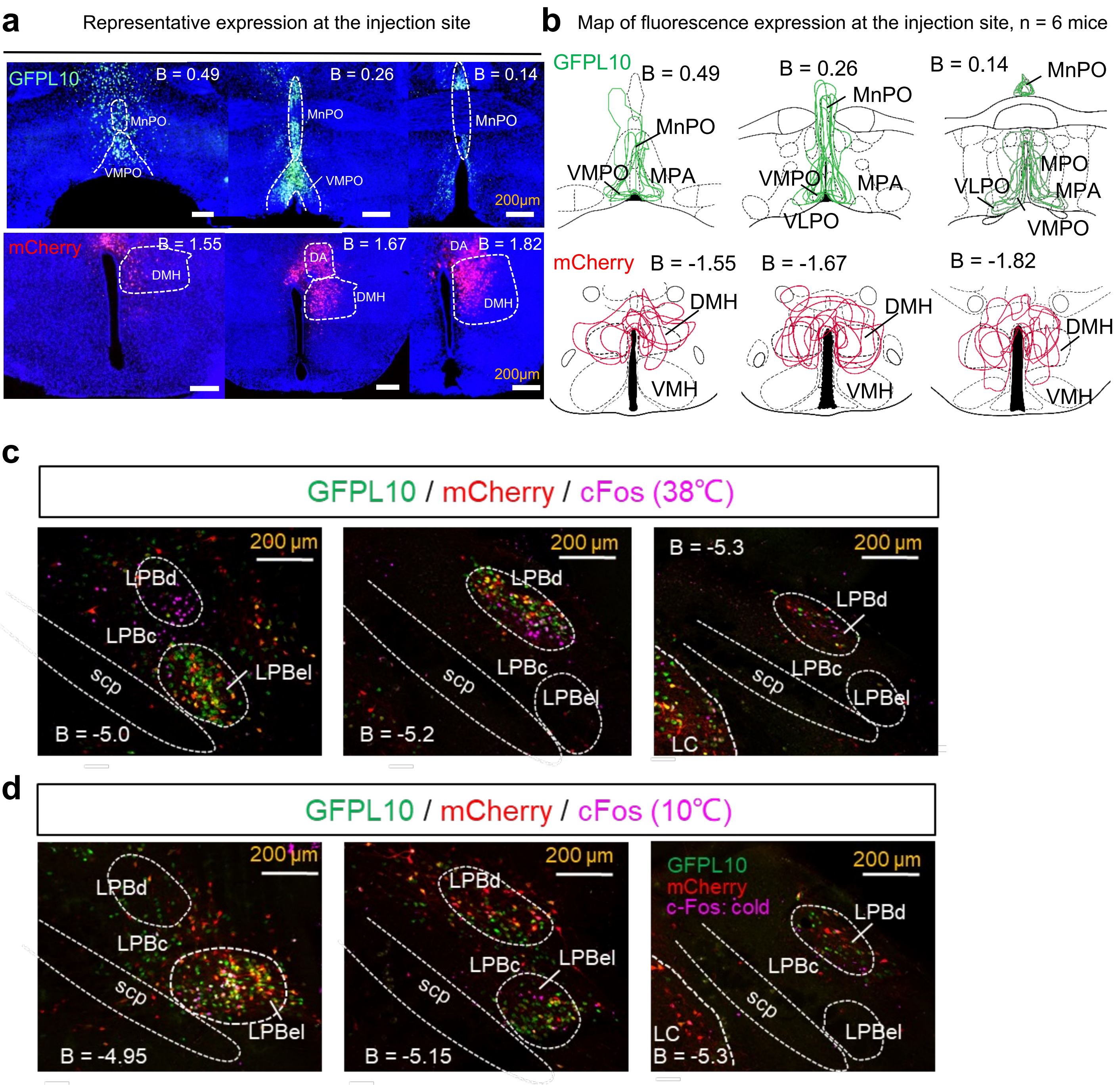

Supplementary Fig. 2 | Mapping the collateral projections of LPB^{Vglut2} neurons to the POA (MnPO & VMPO) and the DMH.

(a) Representative expression of GFP (AAV-Retro-DIO-GFPL10) and mCherry (AAV-Retro-DIO-mCherry) in the injection sites. AAV-Retro-DIO-GFPL10 was injected in the VMPO & MnPO of Vglut2-IRES-Cre mice (n = 6 mice) and AAV-Retro-DIO-mCherry was injected in the DMH of Vglut2-IRES-Cre mice (n = 6 mice). The expression boundaries of each mouse were overlaid in the right as indicated.

(b) Map of fluorescence expression at the injection sites from (a). The expression boundaries of each mouse were overlaid and showed together as indicated (n = 6 mice).

(c) Representative images showing the overlapping between POA- and DMH-projecting LPB^{Vglut2} neurons and warm-activated cFos at different bregma sites. Mice were exposed to warm stimuli (38°C, 2 hrs) before sacrificing for cFos immunostaining. The overlapping ratios were indicated in Fig. 1e. A total of 924 cFos^{+} neurons from 3 mice were scored.

(d) Representative images showing the overlapping between POA/DMH-projecting LPB neurons and cold-activated cFos immunoactivity at different bregma sites. Mice were exposed to cold stimuli (10°C, 2 hrs) before sacrificing for cFos immunostaining. The overlapping ratios were indicated in Fig. 1g. A total of 693 cFos^{+} neurons from 3 mice were scored.

B, bregma; LPBc, lateral parabrachial nucleus, central part; LPBd, lateral parabrachial nucleus, dorsal part; LPBel, lateral parabrachial nucleus, external and internal part; LC, locus coeruleus; scp, superior cerebellar peduncle; DMH, dorsomedial hypothalamus nucleus; DA, dorsal hypothalamus area VMH, ventromedial hypothalamus nucleus; MnPO, median preoptic area; VLPO, ventrolateral preoptic area; MPA, medial preoptic area; MPO, medial preoptic nucleus; VMPO, ventromedial preoptic area.

Supplementary Fig. 3 | Projection pattern of DMH-projecting LPB^{Vglut2} neurons throughout the brain.

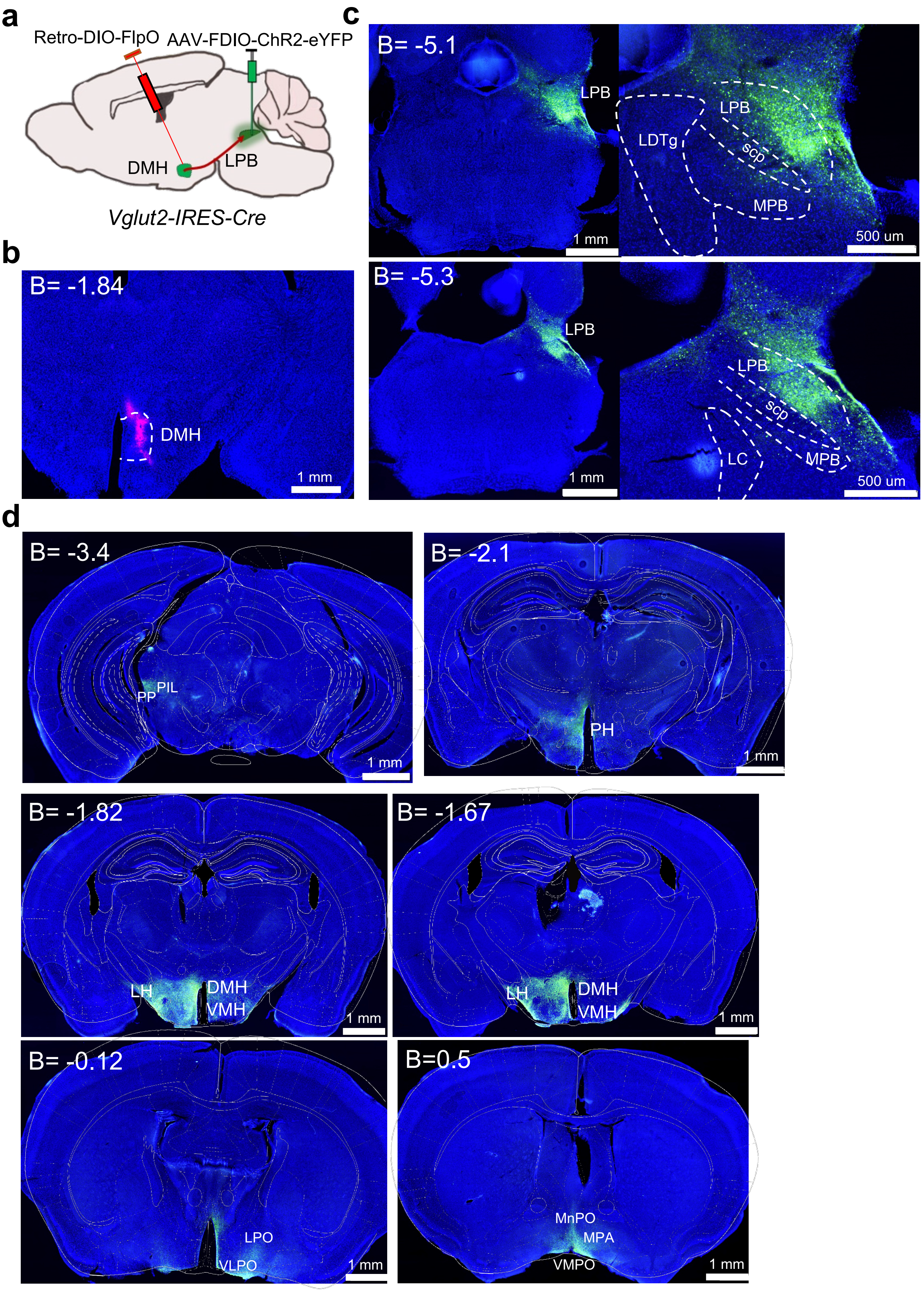

Supplementary Fig. 3 | Projection pattern of DMH-projecting LPB^{Vglut2} neurons throughout the brain.

(a) Scheme for mapping the axonal projections of DMH-projecting LPB^{Vglut2} neurons. For labeling DMH-projecting LPB^{Vglut2} neurons, retrograde AAVs carrying Cre-dependent FlpO were injected in the DMH, which drove the expression of FlpO-dependent ChR2-eYFP in the LPB. A red tracer (CTB647) was co-injected into the DMH to indicate the injection sites.

(b) Representative image showing the injection sites in the DMH viewed by red tracer (CTB647). This experiment was repeated at least 3 times independently with similar results.

(c) Representative images showing ChR2-eYFP expression in DMH-projecting LPB^{Vglut2} neurons in the LPB. This experiment was repeated at least 3 times independently with similar results.

(d) Representative images are showing ChR2-eYFP expression in axonal terminals at various brain sites. This experiment was repeated at least 3 times independently with similar results.

B, bregma; LPB, lateral parabrachial nucleus; LDTg, laterodorsal tegmental nucleus; MPB, medial parabrachial nucleus; LC, locus coeruleus; scp, superior cerebellar peduncle; POA, preoptic nucleus; DMH, dorsomedial hypothalamus; VMH, ventromedial hypothalamus; LH, lateral hypothalamus; PH, poster hypothalamus; LPO, lateral preoptic area; MnPO, median preoptic nucleus; VMPO, ventromedial preoptic nucleus; MPA, medial preoptic area; VLPO, ventrolateral preoptic nucleus; PP, peripeduncular nucleus; PIL, posterior intralaminar thalamic nucleus.

Supplementary Fig. 4 | Phenotypes associated with blocking of DMH^{only}-projecting or POA^{only}-projecting LPB neurons.

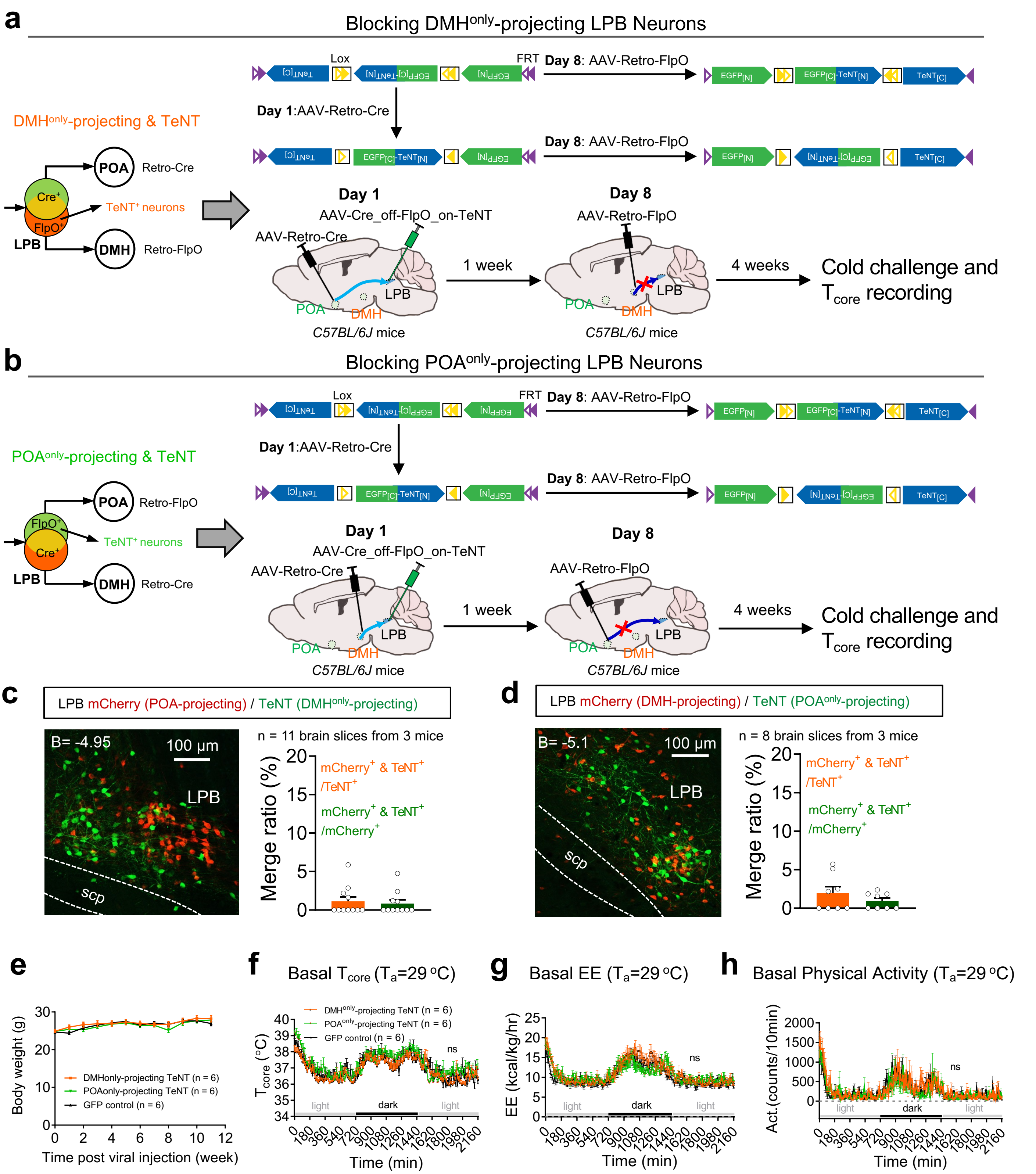

Supplementary Fig. 4 | Phenotypes associated with blocking of DMH^{only}-projecting or POA^{only}-projecting LPB neurons.

(a) The scheme to block LPB neurons that projected to the DMH but did not collaterally project to the POA (DMH^{only}-projecting LPB neurons). On day 1, the retrograde traveling AAV-Retro-hSyn-Cre virus was injected into the POA (MnPO & VMPO) and Cre_off-FlpO_on-TeNT (AAV9-hSyn-Cre_off-FlpO_on-EGFP-T2A-TeNT) injected in the LPB of C57BL/6J mice. Thus, AAV-Retro-Cre would travel to the LPB to turn off TeNT in turning off the TeNT expression in POA-projecting LPB neurons, thus unblocking POA-projecting LPB neurons. On day 8, AAV-Retro-hSyn-FlpO virus was injected into the DMH, which then traveled to the LPB to turn on the TeNT expression in DMH-projecting but not co-projecting LPB neurons (DMH^{only}-projecting LPB neurons). Cre_off-FlpO_on-EGFP (AAV9-hSyn-Cre_off-FlpO_on-EGFP) was used as the control for Cre_off-FlpO_on-TeNT.

(b) The scheme to block LPB neurons that projected to the POA but did not collaterally project to the DMH (POA^{only}-projecting LPB neurons). On day 1, the retrograde traveling AAV-Retro-hSyn-Cre virus was injected into the DMH and Cre_off-FlpO_on-TeNT (AAV9-hSyn-Cre_off-FlpO_on-EGFP-T2A-TeNT) injected in the LPB of C57BL/6J mice. Thus, AAV-Retro-Cre would travel to the LPB to turn off TeNT in turning off the TeNT expression in DMH-projecting LPB neurons, thus unblocking DMH-projecting LPB neurons. On day 8, AAV-Retro-hSyn-FlpO virus was injected into the POA (MnPO & VMPO), which then traveled to the LPB to turn on the TeNT expression in POA-projecting but not co-projecting LPB neurons (POA^{only}-projecting LPB neurons). The same control was used as in (a).

(c) Representative images showing the little overlapping between POA-projecting LPB neurons (mCherry^{+}) and DMH^{only}-projecting LPB neurons (TeNT^{+}). To label the POA-projecting LPB neurons, AAV-hSyn-DIO-mCherry was co-injected with Coff/Fon-TeNT-EGFP in the LPB in (a) so that AAV-Retro-Cre injected in the POA would travel to the LPB to turn on mCherry expression. Scale bar, 100 µm. The overlapping ratios were quantified in the right panel (11 brain slices from 3 mice).

(d) Representative images showing the little overlapping between DMH-projecting LPB neurons (mCherry^{+}) and POA^{only}-projecting LPB neurons (TeNT^{+}). To label the DMH-projecting LPB neurons, AAV-hSyn-DIO-mCherry was co-injected with Coff/Fon-TeNT-EGFP in the LPB in (b) so that AAV-Retro-Cre injected in the DMH would travel to the LPB to turn on mCherry expression. Scale bar, 100 µm. The overlapping ratios were quantified in the right panel (8 brain slices from 3 mice).

(e-h) Changes of body weight (e), basal T_{core} (f), basal energy expenditure (g) and basal physical activity (h) at thermoneutral temperature (f-h) after blocking the DMH^{only}-projecting LPB neurons or POA^{only}-projecting LPB neurons, as indicated (n = 6 mice each).

LPB, lateral parabrachial nucleus; POA, preoptic area; DMH, dorsomedial hypothalamic nucleus; TeNT, Tetanus toxin. All data are shown as mean ± sem. The p-values are calculated based on two-way ANOVA (e-h). ns, not significant. Source data are provided as a Source data file.

Supplementary Fig. 5 | Heatmaps of TeNT expression and baseline measurement after blocking LPB-innervating POA or DMH neurons.

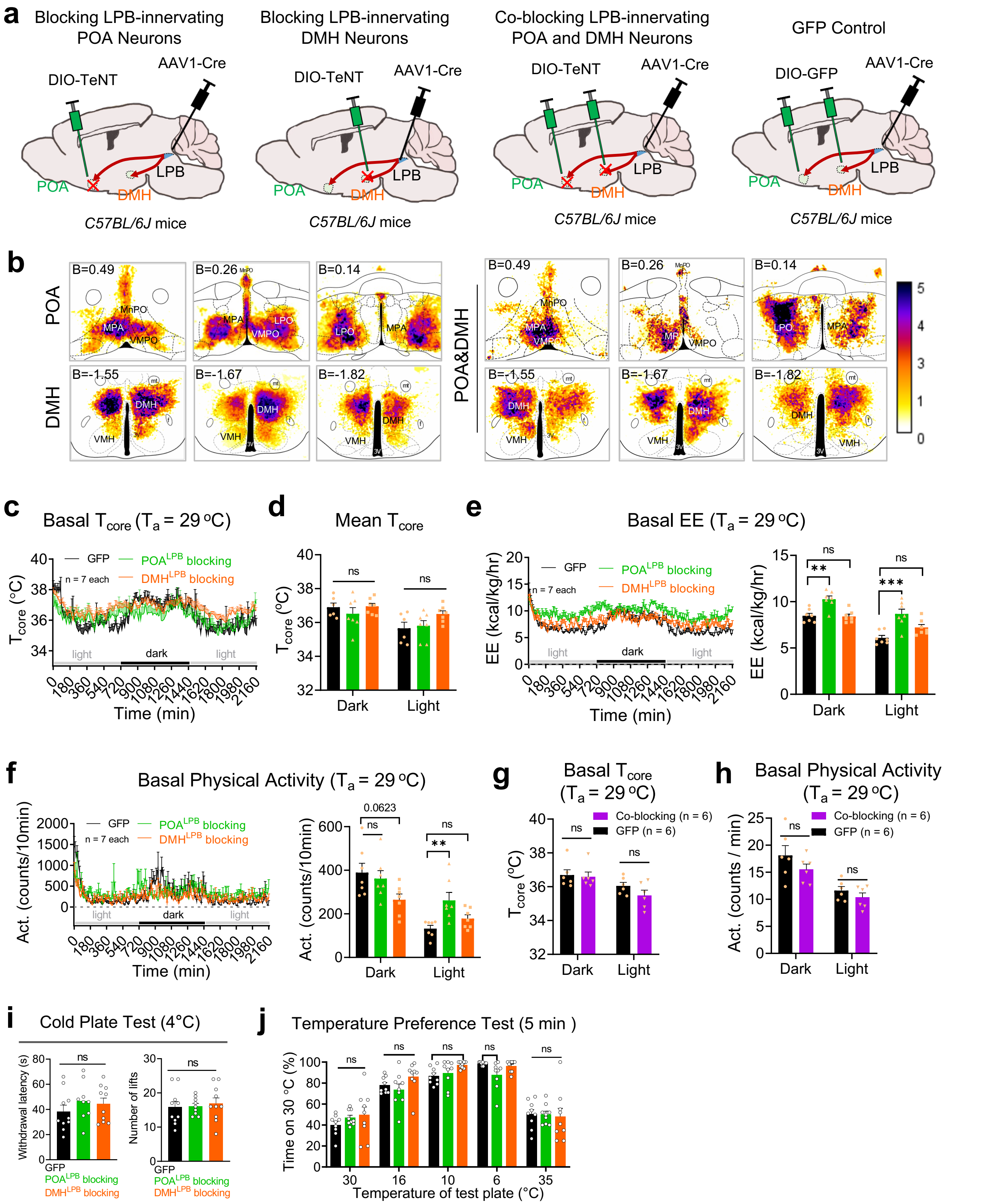

Supplementary Fig. 5 | Heatmaps of TeNT expression and baseline measurement after blocking LPB-innervating POA or DMH neurons.

(a) The scheme to block LPB-innervating POA (mainly MnPO, VMPO, MPA, and LPO) neurons, LPB-innervating DMH neurons, LPB-innervating POA and DMH Neurons (Co-blocking), and GFP control. The anterograde transsynaptic Cre carried by AAV1 (AAV1-hSyn-Cre) was injected in the LPB, which drove the expression of AAV9-hEF1a-DIO-mCherry-P2A-TeNT injected in the POA (POA^{LPB} blocking), the DMH (DMH^{LPB} blocking), or both (co-blocking), as indicated. AAV9-hSyn-DIO-GFP was injected as the control (GFP), where AAV1-hSyn-Cre was injected in the LPB and AAV9-hSyn-DIO-GFP was injected in both the POA and DMH.

(b) Heatmaps of TeNT expression at different Bregma sites from all experimental mice. POA^{LPB} blocking, n = 9 mice; DMH^{LPB} blocking, n = 10 mice; co-blocking, n = 6 mice. The relative scale for the expression intensity (measured by fluorescence intensity) was shown in the right.

(c-f) Changes of basal T_{core} (c-d), basal energy expenditure (e), and basal physical activity (f) at thermoneutral temperature (29°C) after blocking LPB-innervating DMH neurons (DMH^{LPB} blocking) or LPB-innervating POA neurons (POA^{LPB} blocking), as indicated (n = 7 each).

(g-h) Changes of basal T_{core} (g) and basal physical activity (h) at thermoneutral temperature (29°C) after blocking LPB-innervating POA and DMH neurons (Co-blocking), as indicated (n = 6 each).

(i) Withdrawal latency and the number of lifts in the cold plate test (4°C) after blocking LPB-innervating POA/DMH neurons (n = 10 for GFP and DMH^{LPB} blocking group; n = 9 for POA^{LPB} blocking group).

(j) Changes in preference to 30°C versus 35/16/10/6°C after blocking LPB-innervating POA/DMH neurons (n = 9 mice each).

All data are shown as mean ± sem. The p-values are calculated based on one-way ANOVA (d-f, i-j) or unpaired t-test (g-h). The p-values are calculated based on statistical tests in Supplementary Table 2. *p ≤ 0.05; **p ≤ 0.01; ***p ≤ 0.001; ns, not significant. Source data are provided as a Source data file.

Supplementary Fig. 6 | Detailed viral expressions, fiber insert positions, and T_{core} changes of the manipulation of the LPB^{Vglut2}→DMH projection that hit or missed the targets.

a

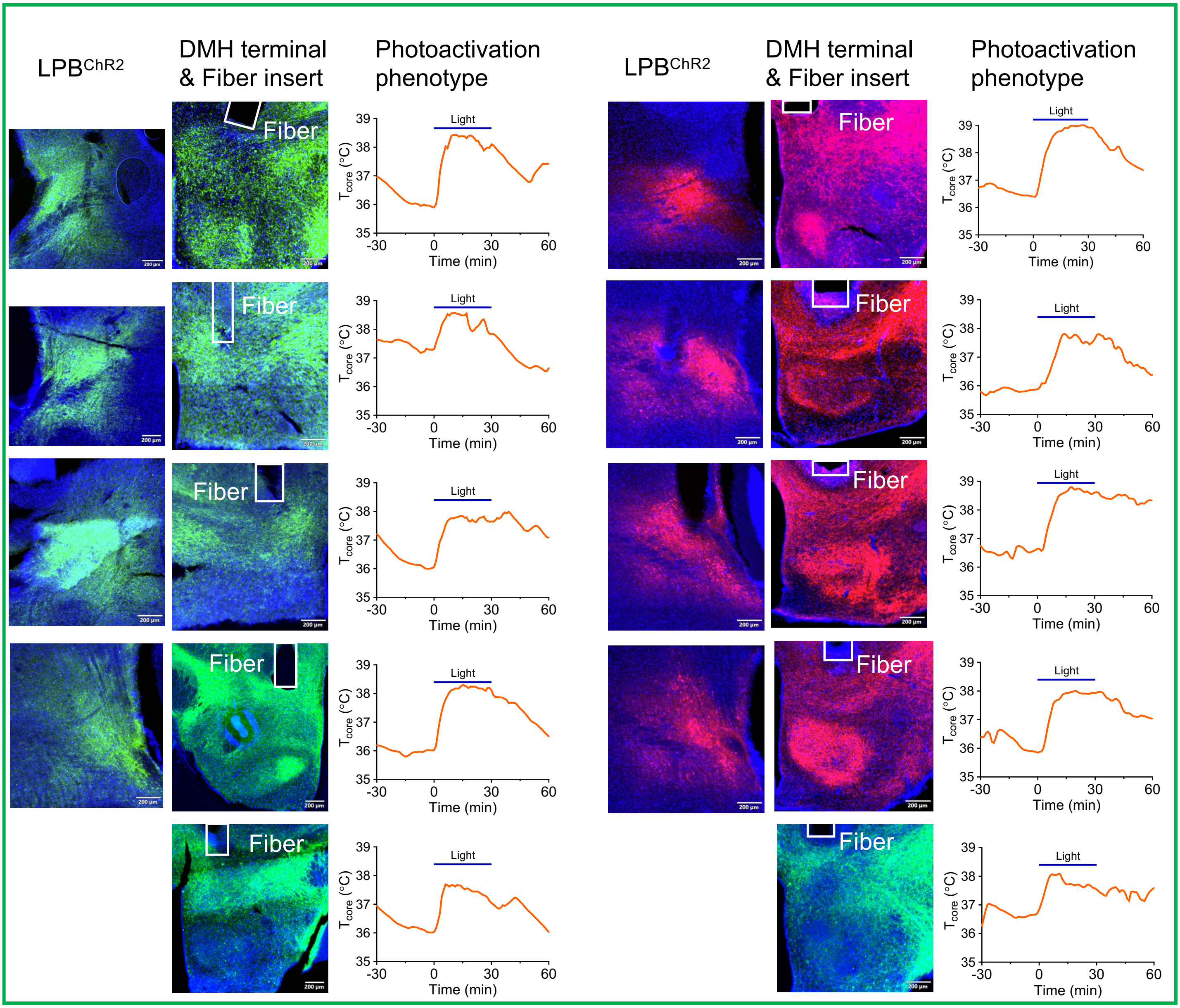

b

One mouse that missed target of photoactivation

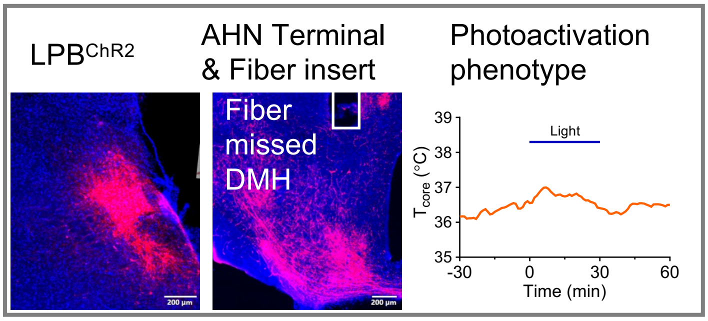

Supplementary Fig. 6 | Detailed viral expressions, fiber insert positions, and T_{core} changes of the manipulation of the LPB^{Vglut2}→DMH projection that hit or missed the targets.

(a) Representative images demonstrating the expression of ChR2 in LPB^{Vglut2} neurons, fiber insert positions and resulting T_{core} changes after photoactivation LPB^{Vglut2}→DMH that hit the target. This experiment was repeated at least 10 times independently with similar results.

(b) Data from one mouse exhibiting missed fiber insert positions and T_{core} changes following photoactivation. AHN, anterior hypothalamic nucleus. This experiment was repeated at least 1 times independently with similar results. Source data are provided as a Source data file.

Supplementary Fig. 7 | Phenotypes associated with neural blocking of POA neurons, DMH^{Vglut2} neurons and DMH^{Vgat} neurons.

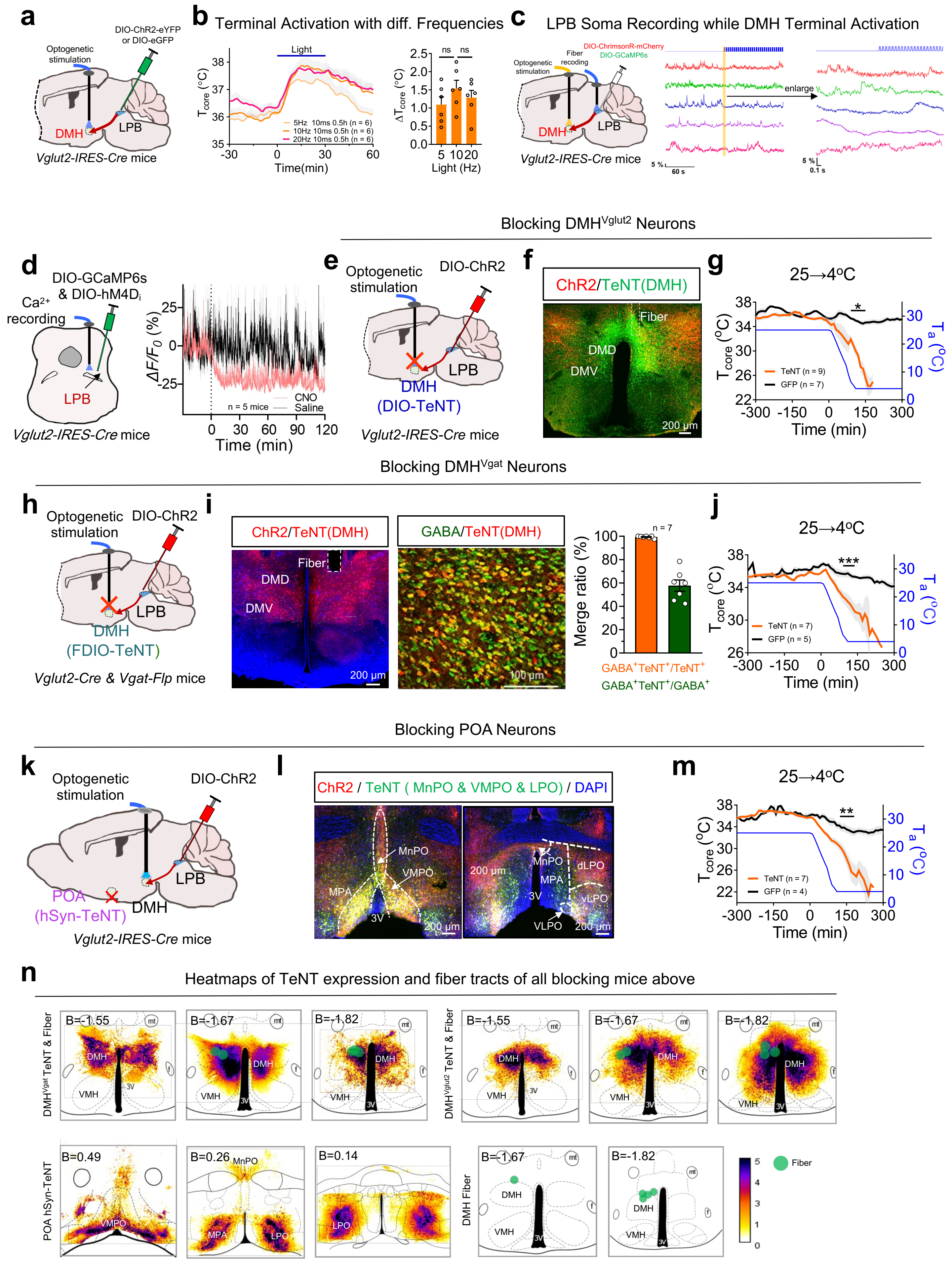

Supplementary Fig. 7 | Phenotypes associated with neural blocking of POA neurons, DMH^{Vglut2} neurons and DMH^{Vgat} neurons.

(a) Scheme of optogenetic activation of glutamatergic (Vglut2+) LPB neural terminals in the DMH.

(b) Changes of T_{core} after 30 min photoactivation of LPB^{Vglut2} neural terminals in the DMH (n = 6 each). Laser pattern: 473 nm, 12mW, Hz as indicated, 30 min.

(c) Scheme for simultaneously expressing the GCaMP6s and ChrimsonR in the LPB, then the DMH terminals were simultaneously activated using a 589nm laser and Ca^{2+} activity was recorded in the LPB (left panel). No obvious calcium signal changes were recorded in LPB after activating DMH's terminals (n = 5 mice). The blue pulse line indicates light stimulation (right panel). Light pattern: 589 nm, 6 mW, 10 Hz, 10 ms, 2-s on followed by 2-s off, with the cycles repeating for 10 min.

(d) Scheme for calcium photometry of LPB^{Vglut2} neurons before and after chemogenetic inhibition. AAVs carrying inhibitory chemogenetic tool hM4D(Gi) (AAV9-EF1α-DIO-hM4D(Gi)-mCherry) and GCaMP6s (AAV9-hSyn-DIO-GCaMP6s) were co-injected in the LPB of Vglut2-Cre mice to block neural activity and record calcium dynamics of LPB^{Vglut2} neurons, respectively. The optical fiber for calcium photometry was implanted in the LPB (left panel).Calcium dynamics of LPB^{Vglut2} neurons before and after chemogenetic inhibition. CNO was injected (i.p., 10mg/kg) at t = 0 min. $\Delta F/F_0$ represents the change in GCaMP6s fluorescence from the mean level (right panel, n = 5 mice).

(e) Scheme for blocking DMH^{Vglut2} neurons using TeNT. AAV5-hSyn-DIO-GFP-P2A-TeNT was injected in the DMH of Vglut2-IRES-Cre mice to block DMH^{Vglut2} neurons.

(f) Representative TeNT expression in the DMH^{Vglut2} neurons. This experiment was repeated at least 9 times independently with similar results.

(g) Changes of T_{core} during cold challenges (25→4°C) after blocking DMH^{Vglut2} neurons (TeNT, n = 9 mice; GFP, n = 7 mice, a total of 10 mice were injected for each group, 1 mouse for TeNT and 3 mice for GFP were excluded from the final analysis due to missed injections or death).

(h) Scheme for blocking DMH^{Vgat} neurons using TeNT. AAV9-hEF1a-FDIO-mCherry-P2A-TeNT was injected in the DMH of Vglut2-IRES-Cre & Vgat-T2A-FlpO mice to block DMH^{Vgat} neurons.

(i) Representative TeNT expression in DMH$^{Vgat\text{-}FlpO}$ neurons and the colocalization of TeNT and GABA immunostaining. The overlapping ratios were quantified in the right. This experiment was repeated at least 7 times independently with similar results.

(j) Changes of T_{core} during cold challenges (25→4°C) after blocking DMH^{Vgat} neurons (TeNT, n = 7 mice; GFP, n = 5 mice, a total of 10 mice for TeNT and 5 mice for GFP were injected for each group, 3 mice for TeNT were excluded from the final analysis due to missed injections or death).

(k) Scheme for blocking POA neurons using TeNT. AAV9-hSyn-eGFP-2A-TeNT were injected in the POA of Vglut2-IRES-Cre mice to block POA neurons.

(l) Representative TeNT expression in the POA (MnPO, VMPO, MPA and LPO) neurons. This experiment was repeated at least 7 times independently with similar results.

(m) Changes of T_{core} during cold challenges (25→4°C) after blocking bulk POA neurons (TeNT, n = 7 mice; GFP, n = 4 mice, a total of 10 mice for TeNT and 5 mice for GFP were injected for each group, 3 mice for TeNT and 1 mouse for GFP were excluded from the final analysis due to missed injections or death).

(n) Heatmaps of TeNT expression and fiber tracts (shown as green dots, each dot represent one mouse) at different Bregma sites from all experimental mice. DMH^{Vglut2} TeNT, n = 9 mice; DMH^{Vgat} TeNT, n = 7 mice; POA TeNT, n = 7 mice. The relative scale for the expression intensity was shown in the right.

All data are shown as mean ± sem. The p-values are calculated based on two-way ANOVA statistical tests. The p-values are calculated based on statistical tests in Supplementary Table 2. $^{**}p \le 0.01$; $^{***}p \le 0.001$. Source data are provided as a Source data file.

Supplementary Fig. 8 | Properties and whole-brain axonal projections of LPB^{SST} neurons.

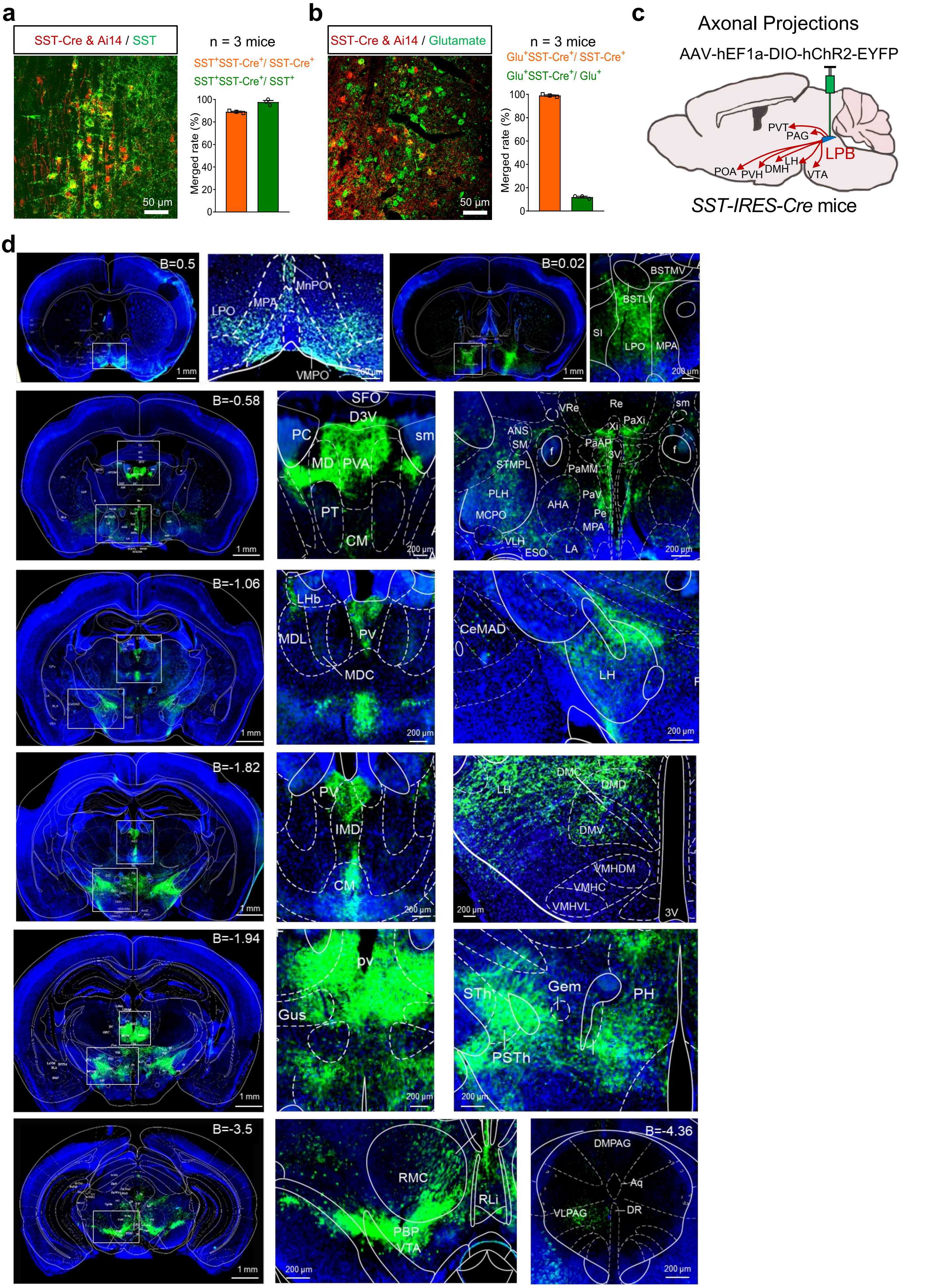

Supplementary Fig. 8 | Properties and whole-brain axonal projections of LPB^{SST} neurons.

(a-b) Overlap between SST-IRES-Cre labeled neurons (Tdt^{+}) in the LPB and the following immuno-positive neurons: SST peptide (a) and glutamate (b). SST-IRES-Cre mice were crossed with Ai14 (Rosa-CAG-LSL-tdTomato-WPRE) to label SST-IRES-Cre in the LPB. Overlap ratios are shown on the right of each panel (n = 3 mice each). All data are shown as mean ± sem. Source data are provided as a Source data file.

(c) The axonal projection pattern of LPB^{SST} neurons throughout the brain, revealed by anterograde tracing AAVs. Injection sites of AAV9-DIO-ChR2-eYFP in the LPB were indicated in the figure. Thicker lines indicated stronger eYFP expressions.

(d) Representative images showing ChR2-eYFP expression in axonal terminals at various brain sites. This experiment was repeated at least 8 times independently with similar results.

AHA, anterior hypothalamic, anterior; B, bregma; BNST, bed nucleus of the stria terminalis; SFO, subfornical organ; sm, stria medullaris of the thalamus; PVA, paraventricular thalamic nucleus, anterior part; PT, paratenia thalamic nucleus; PaAP, paraventricular hypothalamic nucleus, anterior parvic; PaMM, paraventricular hypothalamic nucleus, medial magnocellular part; MDL, mediodorsal thalamic nucleus, lateral part; LHb, lateral habenular nucleus; Pe, periventricular hypothalamic nucleus; PaV, paraventricular hypothalamic nucleus, ventral part; PV, paraventricular thalamic nucleus; BLA, basolateral amygdaloid nucleus, anterior part; CeMAD central amygdaloid nucleus, medial division, anterodorsal part; MD, intermediodorsal thalamic nucleus; DM, central medial thalamic nucleus; PH, posterior hypothalamic nucleus; VMH, ventromedial hypothalamic nucleus; VMHC, ventromedial hypothalamic nucleus, central part; VMHDM, ventromedial hypothalamic nucleus, dorsomedial part; VMHVL, ventromedial hypothalamic nucleus, ventrolateral part; SC, superior colliculus; PSTh parasubthalamic nucleus; STh subthalamic nucleus; POA, preoptic nucleus; Gem, gemini hypothalamic nucleus; Gus gustatory thalamic; VPM, ventral posteromedial thalamic; DMC, dorsomedial hypothalamic nucleus, compact part; DMD, dorsomedial hypothalamic nucleus, dorsal part; RMC, red nucleus, magnocellular part; ZID, zona incerta, dorsal part; Aq, aqueduct(Sylvius); DLPAG, dorsolateral periaqueductal gray; DMPAG, dorsomed periaqueductal; InG, intermediate gray layer of the superior colliculus; LPO, lateral preoptic area; MPA, medial preoptic area; SIB, substantia innominata, basal part; HDB, nucleus of the horizontal limb of the diagonal band; D3V, dorsal 3rd ventricle; PVT, paraventricular thalamic nucleus.

Supplementary Fig. 9 | Phenotypes associated with activation of LPB^{SST} neurons and LPB^{SST}→POA pathway.

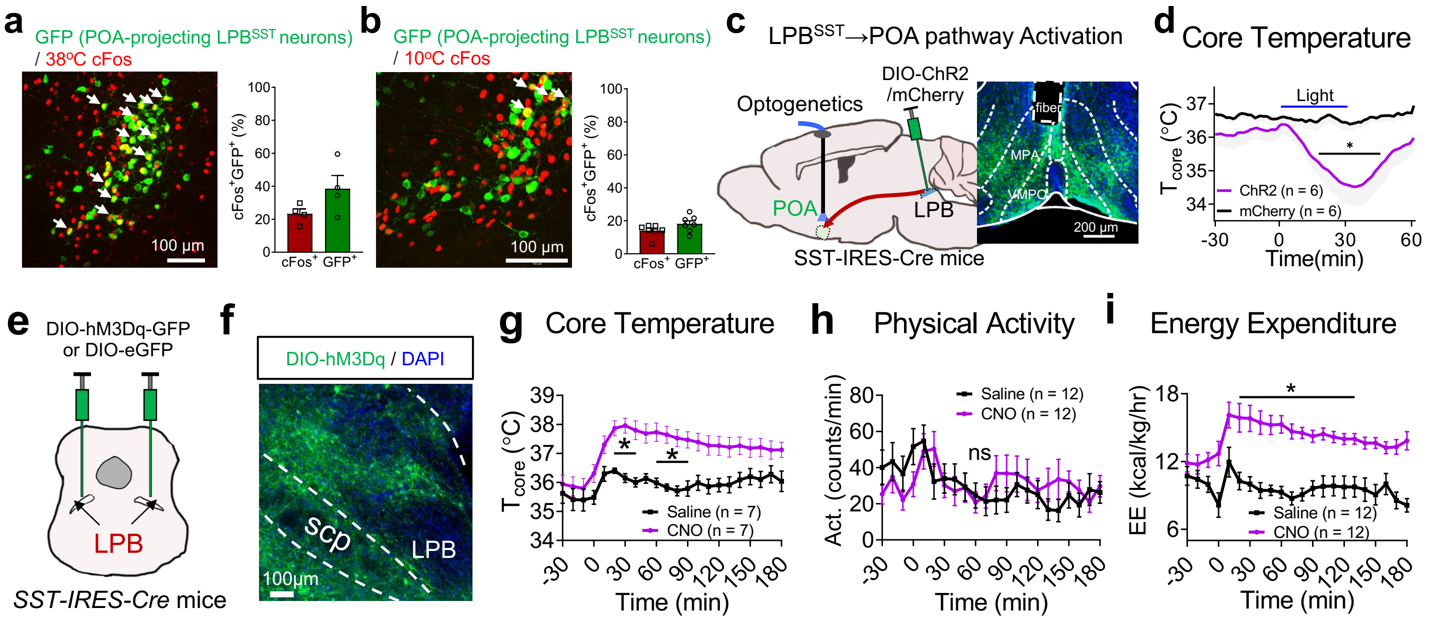

(a-b) Overlap between POA-projecting LPB^{SST} neurons and heat-induced cFos (a) or cold-induced cFos (b) (n = 3 mice each， 1-2 slices for each mouse). To label POA-projecting LPB^{SST} neurons, we injected retrograde AAVs carrying Cre-dependent GFPL10 (AAV-Retro-CAG-Flex-GFPL10) in the VMPO of SST-IRES-Cre mice, which drove the expression of GFPL10 in the LPB. Merged cells were indicated by white arrows.

(c) Design to activate the LPB^{SST}→POA projection via photostimulating of LPB$^{SST\,\&\,ChR2}$ terminals in the VMPO. The representative expression of ChR2-eYFP in the POA is shown in right. This experiment was repeated at least 6 times independently with similar results.

(d) Changes in T_{core} after photoactivation of the LPB^{SST}→POA projection. (n = 6 mice each). Light pattern: 473 nm, 6 mW, 10 Hz, 10 ms, 2-s on followed by 2-s off, with the cycles repeating for 30 min.

(e) Scheme for chemogenetic activation of LPB^{SST} neurons using hM3D$_q$. AAVs carrying hM3D$_q$ (AAV9-hSyn-DIO-hM3D(G$_q$)-eGFP) were injected in the LPB to activate LPB^{SST} neurons.

(f) Representative expression of hM3D$_q$ in LPB^{SST} neurons. This experiment was repeated at least 7 times independently with similar results.

(g-i) Changes of T_{core} (g), physical activity (h), and energy expenditure (i) after chemogenetic activation of LPB^{SST} neurons (n = 12 mice each). CNO was injected (i.p., 2.5 mg/kg) at t = 0 min.

All data are shown as mean ± sem. The p-values are calculated based on RM two-way ANOVA. The p-values are calculated based on statistical tests in Supplementary Table 2. *p ≤ 0.05; ns, not significant. Source data are provided as a Source data file.

Supplementary Fig. 10 | The DMH→RPa pathway is downstream of the LPB→DMH pathway in regulating cold defense.

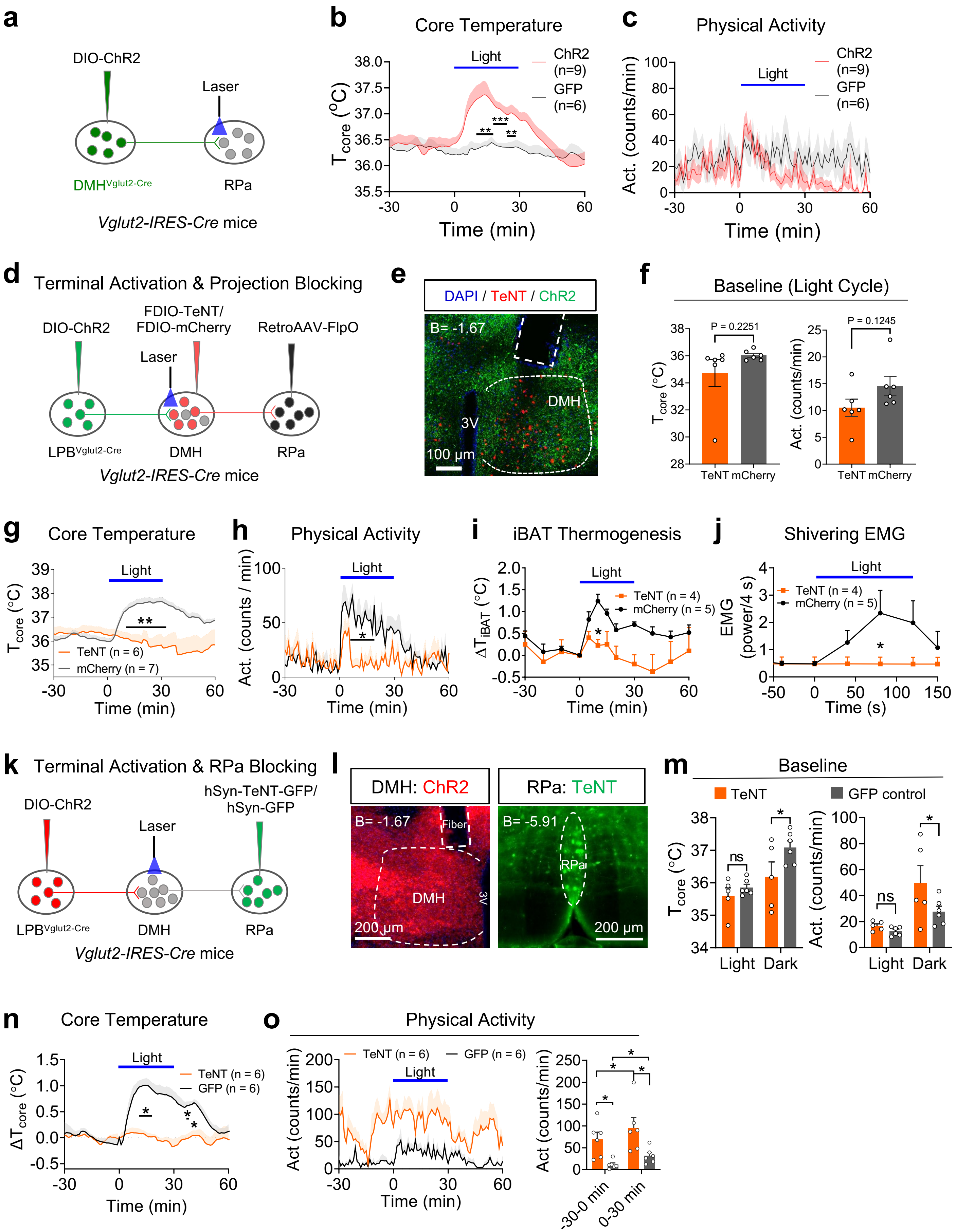

Supplementary Fig. 10 | The DMH→RPa pathway is downstream of the LPB→DMH pathway in regulating cold defense.

(a) Activating the DMH^{Vglut2}→RPa projection via photoactivation of $DMH^{Vglut2\ \&\ ChR2}$ neural terminals in the RPa. AAVs carrying Cre-dependent ChR2 (AAV9-hEF1a-DIO-hChR2-EYFP) were injected into the DMH of Vglut2-IRES-Cre mice. An optical fiber was implanted above the RPa and used for optogenetic activation of neural terminals. AAV9-hSyn-Flex-GFP was used as the control.

(b-c) Changes of T_{core} (b) and physical activity (c) after photoactivation of $DMH^{Vglut2\ \&\ ChR2}$ neural terminals in the RPa (ChR2, n = 9 mice; GFP, n = 6 mice). Light pattern: 473 nm, 12 mW, 10 Hz, 10 ms, 2-s on followed by 2-s off, with the cycles repeating for 30 min.

(d) Photoactivation of LPB^{Vglut2} terminals in the DMH while blocking RPa-projecting DMH neurons. To block RPa-projecting DMH neurons, retrograde AAVs carrying FlpO (AAV-Retro-hSyn-FlpO) were injected in the RPa, which drove the expression of FlpO-dependent TeNT (AAV9-hEF1a-FDIO-mCherry-2A-TeNT) in the DMH.

(e) Representative images of $LPB^{Vglut2\ \&\ ChR2}$ terminals in the DMH (green) and TeNT expression from RPa-projecting DMH neurons (red). This experiment was repeated at least 6 times independently with similar results.

(f) Average basal T_{core} and physical activity after blocking RPa-projecting DMH neurons during the light cycle at T_a = 26 °C (n = 6 mice each).

(g-j) Changes in T_{core} (g), physical activity (h), T_{iBAT} (i), and nuchal muscle EMG (j) in response to photoactivation of LPB^{Vglut2} terminals in the DMH while blocking of RPa-projecting DMH neurons (g-h: TeNT, n = 6 mice; mCherry, n = 7 mice; i-j: TeNT, n = 4 mice; mCherry, n = 5 mice). Light pattern: 473 nm, 6 mW, 10 Hz, 10 ms, 2-s on followed by 2-s off, with the cycles repeating for 30 min in (g-i) and 120 s in (j).

(k) Photoactivation of LPB^{Vglut2} terminals in the DMH while blocking RPa neurons.

(l) Representative images of $LPB^{Vglut2\ \&\ ChR2}$ terminals (red) in the DMH (left panel) and TeNT expression (green) in RPa neurons (right panel). Scale bar, 200 μm. This experiment was repeated at least 6 times independently with similar results.

(m) Average basal T_{core} and physical activity after blocking RPa neurons during the dark and light cycle at T_a = 26 °C (TeNT, n = 5 mice; GFP, n = 6 mice).

(n-o) Changes in ΔT_{core} (n) and physical activity (o) in response to photoactivation of LPB^{Vglut2} terminals in the DMH while blocking of RPa neurons (n = 6 for each group). Light pattern: 473 nm, 6 mW, 10 Hz, 10 ms, 2-s on followed by 2-s off, with the cycles repeating for 30 min. Note, RPa blocking caused compulsive-like circling behavior at the basal level.

LPB, lateral parabrachial nucleus; POA, preoptic area; DMH, dorsomedial hypothalamic nucleus. RPa, raphe pallidus nucleus. All data are the mean ± SEM, (f,m) was analyzed by unpaired t-test, and (b-c, g-j, n-o) were analyzed by two-way RM ANOVA. The p-values are calculated based on statistical tests in Supplementary Table 2. ns, not significant; *p ≤ 0.05 ; **p ≤ 0.01. Source data are provided as a Source data file.

Supplementary Table 1. Summary of TRAP-seq data

gene id	gene name	log2FC	Pvalue	Qvalue
ENSMUSG(	Irgm2	-7.44759	2.14E-10	6.69E-08
ENSMUSG(	Bank1	-6.87598	4.46E-05	0.002274
ENSMUSG(	Ptgr1	-6.47846	1.98E-06	0.000164
ENSMUSG(	Gm7694	-6.46253	2.15E-06	0.000176
ENSMUSG(	Pdzph1	-6.39189	3.85E-05	0.002021
ENSMUSG(	Gm42798	-6.23682	8.28E-05	0.003779
ENSMUSG(	Spi1	-6.19002	6.18E-10	1.68E-07
ENSMUSG(	Pkd2l1	-6.18733	1.08E-07	1.46E-05
ENSMUSG(	Spef2	-6.17151	6.75E-06	0.000471
ENSMUSG(	Nrk	-6.0115	0.000261	0.009605
ENSMUSG(	Mpp4	-5.94979	3.55E-10	1.06E-07
ENSMUSG(	Gm38260	-5.90297	0.000265	0.009693
ENSMUSG(	Pdlim1	-5.82376	4.38E-15	6.13E-12
ENSMUSG(	Fgf2	-5.77165	8.43E-06	0.000559
ENSMUSG(	Igf2	-5.75527	2.45E-08	4.11E-06
ENSMUSG(	Serpinb8	-5.71814	6.70E-05	0.003187
ENSMUSG(	Paqr5	-5.69368	0.000115	0.004956
ENSMUSG(	Pcdhgb8	-5.68369	7.38E-06	0.000504
ENSMUSG(	Ppl	-5.63137	0.001025	0.027926
ENSMUSG(	Eya4	-5.59893	7.89E-06	0.00053
ENSMUSG(	Gm38103	-5.5608	0.000274	0.009949
ENSMUSG(	Echdc3	-5.45174	5.67E-06	0.000407
ENSMUSG(	Hcls1	-5.45124	6.97E-06	0.000482
ENSMUSG(	Gmfg	-5.41435	0.001098	0.029465
ENSMUSG(	Rhod	-5.40237	1.76E-06	0.000149
ENSMUSG(	Cd33	-5.39427	7.41E-05	0.003455
ENSMUSG(	Ppp1r36	-5.36677	0.000886	0.024922
ENSMUSG(	Hey2	-5.34546	6.61E-07	6.54E-05
ENSMUSG(	Tnfaip8l2	-5.31581	3.52E-05	0.00189
ENSMUSG(	Gm37233	-5.30637	0.002285	0.049782
ENSMUSG(	Gm5864	-5.28825	2.68E-08	4.41E-06
ENSMUSG(	Plekhg2	-5.25784	0.002285	0.049782
ENSMUSG(	Car5b	-5.19257	1.85E-05	0.0011
ENSMUSG(	Tmem173	-5.18847	0.000153	0.006322
ENSMUSG(	Bin2	-5.11976	1.45E-07	1.87E-05
ENSMUSG(	Dmbx1	-4.98144	4.52E-07	4.83E-05
ENSMUSG(	Sox14	-4.90977	5.69E-06	0.000408
ENSMUSG(	mt-Tv	-4.90011	1.69E-06	0.000144
ENSMUSG(	Slc32a1	-4.89588	3.89E-07	4.27E-05
ENSMUSG(	Icam1	-4.88452	0.000252	0.009318
ENSMUSG(	Fcer1g	-4.87666	4.53E-08	7.07E-06
ENSMUSG(	Gcnt1	-4.84018	0.000225	0.00854
ENSMUSG(	Hvcn1	-4.82402	7.01E-05	0.003289
ENSMUSG(	Hmgcs2	-4.81701	8.07E-07	7.79E-05
ENSMUSG(	Lrrc74b	-4.81344	0.001643	0.039235
ENSMUSG(	Ocln	-4.79672	5.16E-08	7.82E-06

ENSMUSG(	mt-Tk	-4.76499	8.10E-06	0.000541
ENSMUSG(	Aif1l	-4.75666	4.52E-12	2.38E-09
ENSMUSG(	Slc39a8	-4.7466	3.26E-06	0.00025
ENSMUSG(	Lefty1	-4.74602	0.000387	0.013094
ENSMUSG(	Ecm2	-4.73379	2.50E-06	0.0002
ENSMUSG(	Arap1	-4.68947	9.10E-10	2.32E-07
ENSMUSG(	Ttc23	-4.68796	3.32E-06	0.000253
ENSMUSG(	Iltifb	-4.68095	0.002248	0.04918
ENSMUSG(	Arhgap11a	-4.67462	0.000296	0.010596
ENSMUSG(	Hoxc4	-4.67017	0.000512	0.016105
ENSMUSG(	Tm4sf4	-4.66833	0.000884	0.024922
ENSMUSG(	Sp7	-4.66647	0.000206	0.007986
ENSMUSG(	B230344G1	-4.62213	0.00049	0.015604
ENSMUSG(	P2ry6	-4.60613	0.000317	0.011205
ENSMUSG(	Cd53	-4.59888	5.00E-07	5.22E-05
ENSMUSG(	Lrp2	-4.58444	0.001348	0.034044
ENSMUSG(	Klhl6	-4.56208	0.000144	0.006027
ENSMUSG(	Stard8	-4.55192	9.96E-06	0.000644
ENSMUSG(	Ptprc	-4.54928	9.50E-05	0.004223
ENSMUSG(	Wdr72	-4.5244	0.001238	0.031966
ENSMUSG(	Xlr	-4.50983	0.001154	0.030514
ENSMUSG(	Fcgr3	-4.49788	1.70E-06	0.000144
ENSMUSG(	Tst	-4.49005	9.48E-14	8.96E-11
ENSMUSG(	Sp100	-4.48555	0.001114	0.029647
ENSMUSG(	Gm43201	-4.47573	0.000603	0.018342
ENSMUSG(	Ehd2	-4.45892	7.49E-07	7.32E-05
ENSMUSG(	Fli1	-4.45312	2.98E-05	0.001639
ENSMUSG(	C3ar1	-4.4439	6.35E-05	0.003075
ENSMUSG(	Papss2	-4.43494	8.52E-08	1.17E-05
ENSMUSG(	Crybb1	-4.43079	8.56E-07	8.19E-05
ENSMUSG(	Parp3	-4.4267	8.83E-07	8.35E-05
ENSMUSG(	Clec5a	-4.42595	0.000483	0.015433
ENSMUSG(	Gm43336	-4.41015	0.002107	0.046816
ENSMUSG(	Sds	-4.40776	9.28E-05	0.004152
ENSMUSG(	Uaca	-4.4052	0.000186	0.0074
ENSMUSG(	Adhfe1	-4.40426	9.43E-14	8.96E-11
ENSMUSG(	Slc2a10	-4.3782	5.97E-05	0.002921
ENSMUSG(	mt-Tr	-4.36436	2.70E-09	5.90E-07
ENSMUSG(	Anxa2	-4.36182	1.20E-12	8.02E-10
ENSMUSG(	Chdh	-4.33143	7.21E-06	0.000496
ENSMUSG(	Tagln	-4.32682	1.17E-05	0.000741
ENSMUSG(	Lum	-4.32508	5.12E-06	0.000373
ENSMUSG(	mt-Tg	-4.32118	1.27E-08	2.35E-06
ENSMUSG(	Aif1	-4.32084	6.82E-07	6.72E-05
ENSMUSG(	Ptpn14	-4.32068	3.06E-05	0.00168
ENSMUSG(	Slfn2	-4.2985	6.62E-06	0.000462
ENSMUSG(	Epx	-4.29555	0.000158	0.006478

ENSMUSG(	Kcne1l	-4.28482	3.93E-12	2.10E-09
ENSMUSG(	Ctss	-4.27994	1.77E-14	2.27E-11
ENSMUSG(	Lyz2	-4.25997	2.96E-07	3.44E-05
ENSMUSG(	Acsbg1	-4.24254	5.86E-11	2.30E-08
ENSMUSG(	Lfng	-4.23337	5.16E-07	5.33E-05
ENSMUSG(	Fcrls	-4.208	3.52E-05	0.00189
ENSMUSG(	Myh11	-4.15938	0.000982	0.026961
ENSMUSG(	Pdgfrl	-4.15571	0.001433	0.035487
ENSMUSG(	Grap	-4.15377	1.21E-05	0.000765
ENSMUSG(	Abca9	-4.14629	0.000278	0.010087
ENSMUSG(	Trh	-4.13092	0.000168	0.006802
ENSMUSG(	Lat2	-4.13068	0.000225	0.00854
ENSMUSG(	Gm38309	-4.11985	0.000224	0.008515
ENSMUSG(	mt-Td	-4.11559	0.000135	0.005725
ENSMUSG(	Nat1	-4.10457	4.38E-06	0.000326
ENSMUSG(	Hist1h2bc	-4.10243	6.47E-16	1.83E-12
ENSMUSG(	Acss3	-4.10213	1.41E-05	0.00087
ENSMUSG(	C1qb	-4.09494	4.08E-15	5.95E-12
ENSMUSG(	Pdlim2	-4.09274	3.79E-15	5.79E-12
ENSMUSG(	Abi3	-4.09271	3.25E-06	0.00025
ENSMUSG(	Phgdh	-4.09044	1.35E-16	5.44E-13
ENSMUSG(	S100a13	-4.08811	5.54E-08	8.26E-06
ENSMUSG(	Fam107a	-4.0677	5.02E-13	3.84E-10
ENSMUSG(	Txnip	-4.06504	1.83E-05	0.00109
ENSMUSG(	Gm7901	-4.0647	3.08E-14	3.30E-11
ENSMUSG(	Plin3	-4.06399	1.37E-15	2.90E-12
ENSMUSG(	Fabp7	-4.04284	1.75E-13	1.52E-10
ENSMUSG(	Glis3	-4.04171	0.00053	0.01657
ENSMUSG(	S100a1	-4.03351	2.65E-14	3.16E-11
ENSMUSG(	Gm38299	-4.0092	0.001743	0.040622
ENSMUSG(	Prrx1	-4.00401	3.82E-05	0.00201
ENSMUSG(	Srgn	-4.00108	0.001011	0.02766
ENSMUSG(	Trp63	-3.99354	0.000107	0.004664
ENSMUSG(	Slc9a3r1	-3.99276	7.18E-17	3.30E-13
ENSMUSG(	Irf6	-3.98836	0.001972	0.044717
ENSMUSG(	Trim30a	-3.98581	0.001212	0.031561
ENSMUSG(	Ptpn6	-3.97344	3.91E-06	0.000294
ENSMUSG(	Gm10687	-3.95508	0.002218	0.048776
ENSMUSG(	Acta2	-3.95074	7.87E-07	7.64E-05
ENSMUSG(	Fas	-3.94396	4.94E-05	0.002475
ENSMUSG(	Tmem119	-3.94388	4.50E-06	0.000332
ENSMUSG(	Cldn10	-3.93916	2.71E-12	1.56E-09
ENSMUSG(	Slc25a18	-3.93652	3.35E-15	5.39E-12
ENSMUSG(	Ogn	-3.92133	0.000624	0.018854
ENSMUSG(	Acsf2	-3.90781	7.38E-06	0.000504
ENSMUSG(	Galm	-3.89937	1.64E-05	0.000993
ENSMUSG(	P2ry13	-3.89601	2.95E-05	0.001629

ENSMUSG(	Rfx4	-3.89413	9.83E-10	2.46E-07
ENSMUSG(	mt-Tt	-3.88846	0.000878	0.024851
ENSMUSG(	Sparc	-3.87811	2.23E-12	1.38E-09
ENSMUSG(	Mlc1	-3.8759	4.94E-10	1.41E-07
ENSMUSG(	C1qc	-3.86934	3.68E-12	2.07E-09
ENSMUSG(	C1qa	-3.86136	1.04E-12	7.28E-10
ENSMUSG(	Gm9347	-3.8533	5.29E-08	7.95E-06
ENSMUSG(	Gstt3	-3.85296	1.49E-05	0.000912
ENSMUSG(	mt-Th	-3.84885	4.52E-06	0.000333
ENSMUSG(	Apoe	-3.84371	3.01E-15	5.36E-12
ENSMUSG(	Gm16499	-3.84082	0.001899	0.043445
ENSMUSG(	Mdk	-3.8296	1.83E-08	3.21E-06
ENSMUSG(	Bgn	-3.82395	0.000262	0.009616
ENSMUSG(	Casq2	-3.79815	0.000405	0.013505
ENSMUSG(	Epas1	-3.79405	2.04E-09	4.64E-07
ENSMUSG(	Heph	-3.78933	0.000119	0.005127
ENSMUSG(	Aldh1l1	-3.78707	3.79E-12	2.10E-09
ENSMUSG(	Sox10	-3.78371	9.27E-19	9.93E-15
ENSMUSG(	Itgam	-3.77543	0.000274	0.009949
ENSMUSG(	S100a6	-3.77161	2.43E-05	0.001389
ENSMUSG(	C1qtnf5	-3.76951	4.70E-05	0.002371
ENSMUSG(	Havcr2	-3.76583	0.000121	0.005196
ENSMUSG(	Spin4	-3.76181	0.000609	0.018454
ENSMUSG(	Apod	-3.76074	2.21E-14	2.73E-11
ENSMUSG(	Prob1	-3.76051	0.000241	0.008993
ENSMUSG(	Dio2	-3.75655	2.21E-07	2.66E-05
ENSMUSG(	Zfp820	-3.75478	0.002209	0.048687
ENSMUSG(	Ankub1	-3.74811	0.001743	0.040622
ENSMUSG(	Tns3	-3.74749	1.20E-07	1.60E-05
ENSMUSG(	Ppp1r3g	-3.74298	0.000226	0.008553
ENSMUSG(	Phyhd1	-3.7426	4.57E-07	4.87E-05
ENSMUSG(	Ppp1r14a	-3.7416	1.32E-11	5.96E-09
ENSMUSG(	Plcb3	-3.74093	1.43E-10	4.83E-08
ENSMUSG(	Cdk5rap2	-3.73537	8.28E-11	2.99E-08
ENSMUSG(	Csrp1	-3.72886	2.24E-19	3.60E-15
ENSMUSG(	Klk6	-3.72392	1.12E-09	2.74E-07
ENSMUSG(	mt-Rnr2	-3.72289	1.80E-10	5.80E-08
ENSMUSG(	Foxb1	-3.71745	0.000282	0.010218
ENSMUSG(	Klrg2	-3.71114	4.25E-05	0.002187
ENSMUSG(	Slc14a1	-3.70992	2.28E-12	1.38E-09
ENSMUSG(	Msx2	-3.70765	0.000701	0.020855
ENSMUSG(	Lad1	-3.69041	0.001475	0.036243
ENSMUSG(	Olfml3	-3.69022	2.46E-08	4.11E-06
ENSMUSG(	Acsm5	-3.6895	0.001391	0.034848
ENSMUSG(	Olig1	-3.68944	6.50E-18	4.18E-14
ENSMUSG(	Gm4524	-3.68519	0.000216	0.008287
ENSMUSG(	mt-Tl2	-3.68501	4.14E-05	0.002145

ENSMUSG(	Myo1f	-3.67704	0.001006	0.027552
ENSMUSG(	Dock5	-3.67131	0.001107	0.02956
ENSMUSG(	mt-Nd3	-3.65552	7.36E-11	2.78E-08
ENSMUSG(	Col1a2	-3.64275	2.50E-05	0.001424
ENSMUSG(	Gng12	-3.63843	3.06E-14	3.30E-11
ENSMUSG(	Tor4a	-3.63543	0.002034	0.045519
ENSMUSG(	Aldh1a1	-3.63427	3.00E-14	3.30E-11
ENSMUSG(	Dbx2	-3.62724	3.92E-12	2.10E-09
ENSMUSG(	Satb2	-3.62123	0.001069	0.028783
ENSMUSG(	mt-Tl1	-3.61242	7.74E-09	1.53E-06
ENSMUSG(	Car2	-3.61075	9.79E-18	5.24E-14
ENSMUSG(	Gstk1	-3.60182	1.18E-06	0.000105
ENSMUSG(	Etnppl	-3.59231	3.79E-11	1.58E-08
ENSMUSG(	Pycard	-3.59054	4.45E-05	0.002273
ENSMUSG(	Qprt	-3.57754	0.001222	0.031711
ENSMUSG(	Laptm5	-3.57439	1.57E-10	5.16E-08
ENSMUSG(	Tek	-3.57081	0.000337	0.011711
ENSMUSG(	mt-Ti	-3.56327	1.28E-06	0.000112
ENSMUSG(	Cd74	-3.55965	3.40E-05	0.001838
ENSMUSG(	Fblim1	-3.55334	0.001353	0.034119
ENSMUSG(	Pdlim4	-3.55227	4.50E-06	0.000332
ENSMUSG(	Hsd11b1	-3.55048	5.31E-05	0.002634
ENSMUSG(	Atp1a2	-3.5476	1.04E-12	7.28E-10
ENSMUSG(	AC167565.	-3.54589	0.000293	0.010541
ENSMUSG(	Sdc4	-3.5429	1.35E-12	8.69E-10
ENSMUSG(	Gas2l3	-3.53988	0.000619	0.018714
ENSMUSG(	Slc6a11	-3.53367	6.08E-12	3.05E-09
ENSMUSG(	Art3	-3.53191	6.76E-05	0.003204
ENSMUSG(	Samd9l	-3.52374	0.000324	0.011376
ENSMUSG(	Litaf	-3.52345	3.70E-16	1.19E-12
ENSMUSG(	E130114P1	-3.52084	1.41E-06	0.000122
ENSMUSG(	Gpr17	-3.52048	0.000298	0.010644
ENSMUSG(	Kcnj10	-3.51706	1.80E-10	5.80E-08
ENSMUSG(	Oaf	-3.51622	0.000458	0.014896
ENSMUSG(	Mpeg1	-3.51579	0.000209	0.008054
ENSMUSG(	Serpinb1a	-3.50633	2.06E-13	1.69E-10
ENSMUSG(	BC064078	-3.50544	0.001871	0.043031
ENSMUSG(	Gsdmd	-3.50533	0.000449	0.014647
ENSMUSG(	Bbox1	-3.50303	9.96E-12	4.78E-09
ENSMUSG(	Scrg1	-3.50077	3.21E-07	3.66E-05
ENSMUSG(	Ly86	-3.49386	5.19E-06	0.000376
ENSMUSG(	Xdh	-3.48567	0.000206	0.007986
ENSMUSG(	2310022B(	-3.48544	1.05E-10	3.70E-08
ENSMUSG(	Hist1h2bg	-3.48518	1.40E-07	1.84E-05
ENSMUSG(	Gad1	-3.48197	3.38E-13	2.65E-10
ENSMUSG(	Dock8	-3.46541	6.80E-05	0.003214
ENSMUSG(	Pdk4	-3.46119	4.34E-06	0.000324

ENSMUSG(	Slc1a3	-3.45684	2.39E-11	1.01E-08
ENSMUSG(	Cd38	-3.45088	0.000438	0.014396
ENSMUSG(	Gbp2	-3.44495	2.15E-05	0.001241
ENSMUSG(	Ncf2	-3.44457	1.33E-05	0.000833
ENSMUSG(	Gpr37l1	-3.44308	7.96E-11	2.96E-08
ENSMUSG(	Plekha2	-3.43277	2.18E-09	4.93E-07
ENSMUSG(	2900052N(	-3.43197	1.06E-08	2.01E-06
ENSMUSG(	Npr3	-3.43124	0.000447	0.01461
ENSMUSG(	Fa2h	-3.43	8.24E-10	2.15E-07
ENSMUSG(	Xaf1	-3.42626	8.80E-06	0.000578
ENSMUSG(	Cyp2j9	-3.4221	2.51E-09	5.61E-07
ENSMUSG(	Lcat	-3.41777	1.63E-08	2.94E-06
ENSMUSG(	Ermn	-3.41728	1.43E-15	2.90E-12
ENSMUSG(	Dao	-3.40711	0.001223	0.031721
ENSMUSG(	Acss1	-3.40434	8.51E-08	1.17E-05
ENSMUSG(	Gfap	-3.40138	1.06E-12	7.28E-10
ENSMUSG(	Ifi35	-3.40035	2.88E-05	0.001603
ENSMUSG(	Ramp1	-3.39622	1.14E-10	3.94E-08
ENSMUSG(	Ninj2	-3.39119	4.24E-05	0.002187
ENSMUSG(	Bdh2	-3.3882	4.33E-07	4.66E-05
ENSMUSG(	Rarres2	-3.38748	0.001757	0.040839
ENSMUSG(	Inppl1	-3.38741	1.34E-09	3.22E-07
ENSMUSG(	Slc25a34	-3.38648	9.43E-07	8.84E-05
ENSMUSG(	Mgst1	-3.38601	1.12E-09	2.74E-07
ENSMUSG(	Cyp2j12	-3.37985	6.45E-06	0.000452
ENSMUSG(	Rftn2	-3.37663	9.03E-09	1.76E-06
ENSMUSG(	Axl	-3.3729	2.03E-10	6.39E-08
ENSMUSG(	Arhgap45	-3.36944	0.000512	0.016104
ENSMUSG(	Irf8	-3.36879	0.000359	0.012316
ENSMUSG(	Pla2g16	-3.36867	3.94E-14	4.08E-11
ENSMUSG(	Tyrobp	-3.36648	0.000205	0.007986
ENSMUSG(	Gm28438	-3.35249	5.10E-08	7.81E-06
ENSMUSG(	Rbm12b1	-3.3443	3.62E-05	0.001936
ENSMUSG(	Plekhf1	-3.34286	7.55E-10	1.99E-07
ENSMUSG(	A930017K1	-3.34049	0.000488	0.015561
ENSMUSG(	Olfr287	-3.32926	7.72E-06	0.000521
ENSMUSG(	A330076C(	-3.31542	0.000899	0.025193
ENSMUSG(	Pxn	-3.31535	3.48E-08	5.62E-06
ENSMUSG(	Pcp2	-3.30621	3.44E-07	3.89E-05
ENSMUSG(	Agt	-3.30479	3.35E-09	7.17E-07
ENSMUSG(	Fgfr3	-3.29733	6.82E-16	1.83E-12
ENSMUSG(	Pax3	-3.29383	0.00036	0.012321
ENSMUSG(	Efhd1	-3.29158	8.88E-08	1.21E-05
ENSMUSG(	Sox8	-3.29034	1.02E-11	4.80E-09
ENSMUSG(	mt-Nd4l	-3.28833	9.78E-10	2.46E-07
ENSMUSG(	Cyp4v3	-3.28395	1.40E-05	0.000868
ENSMUSG(	Cldn11	-3.28116	1.53E-06	0.000132

ENSMUSG(	Slc38a3	-3.27977	2.19E-07	2.64E-05
ENSMUSG(	Lef1	-3.27819	0.000976	0.026843
ENSMUSG(	Selplg	-3.27411	5.68E-11	2.25E-08
ENSMUSG(	Heyl	-3.2741	3.62E-10	1.07E-07
ENSMUSG(	Trim34a	-3.27323	0.001109	0.029588
ENSMUSG(	Selenop	-3.2728	1.04E-12	7.28E-10
ENSMUSG(	Lhx5	-3.27133	2.03E-06	0.000166
ENSMUSG(	Lhx1os	-3.2692	7.15E-07	7.01E-05
ENSMUSG(	Il33	-3.26606	1.85E-13	1.56E-10
ENSMUSG(	Suclg2	-3.26578	3.93E-08	6.22E-06
ENSMUSG(	Wipf1	-3.26217	3.71E-07	4.11E-05
ENSMUSG(	Fmo1	-3.2604	6.90E-08	9.85E-06
ENSMUSG(	Mylk	-3.25355	8.15E-06	0.000543
ENSMUSG(	Mal	-3.24094	5.64E-12	2.88E-09
ENSMUSG(	Epb41l2	-3.23759	6.25E-05	0.003038
ENSMUSG(	Timp4	-3.23283	6.42E-08	9.21E-06
ENSMUSG(	Plp1	-3.23229	9.14E-09	1.77E-06
ENSMUSG(	Trim56	-3.23218	0.000378	0.012854
ENSMUSG(	Dapp1	-3.22606	0.000283	0.010227
ENSMUSG(	Wwtr1	-3.2151	0.000321	0.011296
ENSMUSG(	Slc7a10	-3.21359	1.98E-07	2.46E-05
ENSMUSG(	Sox5	-3.20862	7.60E-09	1.52E-06
ENSMUSG(	Agmo	-3.2065	0.000241	0.008976
ENSMUSG(	Htra3	-3.20258	0.000265	0.009695
ENSMUSG(	BC026585	-3.19932	2.50E-08	4.15E-06
ENSMUSG(	Nid1	-3.19859	0.000433	0.014284
ENSMUSG(	Gpd1	-3.1931	1.06E-11	4.95E-09
ENSMUSG(	Folh1	-3.18633	1.47E-07	1.88E-05
ENSMUSG(	Wnt7a	-3.18532	0.00024	0.008976
ENSMUSG(	Ctdsp2	-3.18419	1.24E-08	2.30E-06
ENSMUSG(	Copz2	-3.18076	1.48E-06	0.000128
ENSMUSG(	Igfbp2	-3.17607	0.000125	0.00533
ENSMUSG(	Hist1h2be	-3.17536	1.45E-05	0.000891
ENSMUSG(	Opalin	-3.17339	3.08E-09	6.63E-07
ENSMUSG(	Entpd1	-3.17122	0.001428	0.035436
ENSMUSG(	Rgs5	-3.16887	0.000188	0.007448
ENSMUSG(	Clic1	-3.16353	5.99E-07	5.99E-05
ENSMUSG(	Paqr6	-3.15851	2.60E-07	3.08E-05
ENSMUSG(	Lhx1	-3.15815	0.001324	0.033617
ENSMUSG(	Ndrg2	-3.15318	9.10E-12	4.43E-09
ENSMUSG(	Vcam1	-3.14585	4.80E-11	1.93E-08
ENSMUSG(	Hpgds	-3.14496	1.42E-05	0.000871
ENSMUSG(	Hepacam	-3.13986	1.13E-11	5.18E-09
ENSMUSG(	Ifitm3	-3.13217	0.000234	0.008796
ENSMUSG(	Zfp385c	-3.12855	0.00146	0.035975
ENSMUSG(	Csf1r	-3.11744	4.81E-07	5.10E-05
ENSMUSG(	Aqp4	-3.11544	1.65E-14	2.21E-11

ENSMUSG(	Gpt	-3.11455	5.21E-09	1.08E-06
ENSMUSG(	Aldh3b1	-3.10808	1.24E-05	0.000781
ENSMUSG(	Gjb2	-3.1056	7.78E-06	0.000524
ENSMUSG(	Tmem63a	-3.10553	3.14E-06	0.000244
ENSMUSG(	Tgfbi	-3.10408	0.00167	0.039558
ENSMUSG(	Myl9	-3.09825	1.01E-07	1.37E-05
ENSMUSG(	Pax6	-3.09798	9.09E-05	0.004096
ENSMUSG(	Tmbim1	-3.09714	1.06E-10	3.72E-08
ENSMUSG(	Slc7a7	-3.09317	0.001043	0.028328
ENSMUSG(	Bfsp2	-3.09145	2.25E-06	0.000182
ENSMUSG(	Gja1	-3.08944	1.36E-09	3.23E-07
ENSMUSG(	C030029H(	-3.08337	0.001995	0.044918
ENSMUSG(	Ucp3	-3.07302	1.90E-05	0.001122
ENSMUSG(	mt-Tq	-3.0726	0.000484	0.015473
ENSMUSG(	Rgl3	-3.07258	0.000934	0.025896
ENSMUSG(	Sparcl1	-3.0657	8.01E-11	2.96E-08
ENSMUSG(	Sardh	-3.06529	0.001049	0.028415
ENSMUSG(	Vsir	-3.06477	5.10E-05	0.002545
ENSMUSG(	2700046A(	-3.05735	0.001857	0.042775
ENSMUSG(	S100b	-3.05698	1.79E-12	1.12E-09
ENSMUSG(	Pls1	-3.05258	1.02E-08	1.95E-06
ENSMUSG(	Adamtsl4	-3.05149	0.000838	0.024087
ENSMUSG(	Sult1a1	-3.0492	9.18E-07	8.65E-05
ENSMUSG(	Nkx2-2	-3.04712	0.000295	0.010568
ENSMUSG(	mt-Rnr1	-3.04636	8.36E-08	1.16E-05
ENSMUSG(	Aspa	-3.04605	9.98E-11	3.56E-08
ENSMUSG(	Irak3	-3.04145	0.001402	0.035029
ENSMUSG(	Egfr	-3.04071	0.001271	0.0326
ENSMUSG(	Plekho2	-3.0321	1.10E-08	2.07E-06
ENSMUSG(	Spag5	-3.03205	6.78E-06	0.000472
ENSMUSG(	Plekhh1	-3.02872	6.60E-05	0.003155
ENSMUSG(	Sfrp5	-3.02127	4.96E-07	5.20E-05
ENSMUSG(	Ezh2	-3.01953	6.66E-05	0.003171
ENSMUSG(	Tifa	-3.0159	1.05E-06	9.61E-05
ENSMUSG(	S1pr1	-3.01465	4.22E-11	1.72E-08
ENSMUSG(	Ctsh	-3.01046	5.28E-06	0.000382
ENSMUSG(	Sox9	-3.01034	1.44E-09	3.41E-07
ENSMUSG(	Kcnj16	-3.00866	6.39E-10	1.71E-07
ENSMUSG(	Rell1	-3.004	0.000776	0.02258
ENSMUSG(	Cyba	-3.00355	2.12E-05	0.001229
ENSMUSG(	Carhsp1	-3.00197	4.78E-10	1.37E-07
ENSMUSG(	Id3	-3.00101	8.57E-10	2.20E-07
ENSMUSG(	Cideb	-2.99609	0.002186	0.048314
ENSMUSG(	Tnfrsf1a	-2.99608	4.07E-05	0.002116
ENSMUSG(	Abca1	-2.9923	4.94E-07	5.19E-05
ENSMUSG(	Slc1a2	-2.98986	2.67E-09	5.87E-07
ENSMUSG(	Hhip	-2.98825	0.000266	0.009739

ENSMUSG(	S100a16	-2.98739	3.93E-10	1.15E-07
ENSMUSG(	Mfge8	-2.98346	3.88E-07	4.27E-05
ENSMUSG(	Ddit4l	-2.97469	0.002006	0.045075
ENSMUSG(	Dock1	-2.97114	5.89E-08	8.64E-06
ENSMUSG(	Il17ra	-2.97085	0.000137	0.00579
ENSMUSG(	Gm40578	-2.94781	0.000214	0.00822
ENSMUSG(	Pdgfra	-2.94623	0.001233	0.031889
ENSMUSG(	Lgi4	-2.94159	2.50E-06	0.0002
ENSMUSG(	Itih5	-2.93626	3.04E-08	4.93E-06
ENSMUSG(	Arhgef10	-2.93579	6.81E-09	1.38E-06
ENSMUSG(	Itih3	-2.9306	1.31E-07	1.74E-05
ENSMUSG(	Calca	-2.93045	6.02E-08	8.75E-06
ENSMUSG(	Slc30a3	-2.92849	8.13E-08	1.13E-05
ENSMUSG(	Dennd4c	-2.92663	0.000924	0.025713
ENSMUSG(	Itgb4	-2.92619	0.002104	0.046798
ENSMUSG(	Gm10222	-2.92514	2.11E-07	2.57E-05
ENSMUSG(	Sfxn5	-2.92138	9.87E-10	2.46E-07
ENSMUSG(	Fcgrt	-2.92084	8.04E-08	1.12E-05
ENSMUSG(	Ccdc190	-2.92074	0.002044	0.045651
ENSMUSG(	Gatsl3	-2.91787	1.34E-05	0.000834
ENSMUSG(	Slc39a12	-2.91388	1.17E-09	2.84E-07
ENSMUSG(	Hadh	-2.91336	5.29E-12	2.74E-09
ENSMUSG(	Acvrl1	-2.91058	0.001274	0.0326
ENSMUSG(	Dmp1	-2.90913	0.001665	0.039447
ENSMUSG(	Htra1	-2.90392	1.03E-06	9.45E-05
ENSMUSG(	Cyp4f14	-2.90199	9.72E-06	0.000631
ENSMUSG(	Plekhg3	-2.89968	9.55E-09	1.84E-06
ENSMUSG(	Phkg1	-2.89683	7.27E-08	1.03E-05
ENSMUSG(	Mro	-2.89643	8.67E-07	8.27E-05
ENSMUSG(	Tmem100	-2.89611	3.65E-09	7.77E-07
ENSMUSG(	Cmtm5	-2.88284	5.25E-10	1.46E-07
ENSMUSG(	Elovl7	-2.88234	1.13E-07	1.51E-05
ENSMUSG(	Mog	-2.87907	3.57E-08	5.74E-06
ENSMUSG(	Lyn	-2.87701	0.00201	0.045147
ENSMUSG(	Pygm	-2.87596	0.000355	0.012194
ENSMUSG(	Amt	-2.87509	0.000169	0.006843
ENSMUSG(	Anln	-2.87248	4.75E-10	1.37E-07
ENSMUSG(	Magt1	-2.87105	1.16E-06	0.000103
ENSMUSG(	Mboat1	-2.87059	0.00193	0.043989
ENSMUSG(	mt-Tm	-2.86575	0.000802	0.023166
ENSMUSG(	Hist1h1c	-2.8646	1.30E-07	1.73E-05
ENSMUSG(	Rlbp1	-2.86445	1.37E-05	0.000852
ENSMUSG(	Vtn	-2.86255	4.91E-07	5.19E-05
ENSMUSG(	Gpsm2	-2.85224	0.000521	0.016301
ENSMUSG(	Gm10644	-2.84985	0.001643	0.039235
ENSMUSG(	Homer3	-2.84911	8.78E-07	8.32E-05
ENSMUSG(	Igsf11	-2.84847	0.000227	0.008595

ENSMUSG(	Cx3cr1	-2.8481	0.000428	0.014152
ENSMUSG(	Prss35	-2.84767	0.000206	0.007986
ENSMUSG(	Colec12	-2.84349	0.002266	0.049444
ENSMUSG(	Snap23	-2.84145	1.17E-05	0.000744
ENSMUSG(	Padi2	-2.83873	5.15E-10	1.45E-07
ENSMUSG(	Ppp1r1b	-2.83446	1.47E-11	6.38E-09
ENSMUSG(	Gstm2-ps1	-2.83279	5.28E-10	1.46E-07
ENSMUSG(	Gng11	-2.82817	6.40E-09	1.32E-06
ENSMUSG(	Naprt	-2.82593	0.000114	0.004927
ENSMUSG(	Pctp	-2.82581	8.71E-06	0.000574
ENSMUSG(	Gm14204	-2.82549	8.04E-05	0.00368
ENSMUSG(	Cp	-2.817	0.001909	0.043604
ENSMUSG(	Tmem51	-2.81649	0.00171	0.040136
ENSMUSG(	A2m	-2.81597	0.000165	0.00671
ENSMUSG(	Gstm1	-2.81291	2.48E-12	1.45E-09
ENSMUSG(	Rbp1	-2.81251	0.000172	0.006942
ENSMUSG(	Lmcd1	-2.81174	3.02E-07	3.49E-05
ENSMUSG(	Ttyh2	-2.81118	6.22E-07	6.19E-05
ENSMUSG(	Slc17a7	-2.80961	6.05E-06	0.000429
ENSMUSG(	Gm8834	-2.80838	3.47E-05	0.001869
ENSMUSG(	Prkd3	-2.80835	6.13E-07	6.12E-05
ENSMUSG(	Slc6a5	-2.80513	9.12E-05	0.004105
ENSMUSG(	Rhog	-2.80473	1.48E-09	3.48E-07
ENSMUSG(	Tnfaip6	-2.80262	3.23E-07	3.67E-05
ENSMUSG(	Cpt1a	-2.79996	2.43E-06	0.000195
ENSMUSG(	Gm5637	-2.79653	0.001783	0.041304
ENSMUSG(	Tmem98	-2.79634	5.34E-07	5.49E-05
ENSMUSG(	Ddx58	-2.79622	0.00025	0.009265
ENSMUSG(	Metrn	-2.79152	2.31E-08	3.93E-06
ENSMUSG(	Glul	-2.78463	7.07E-12	3.50E-09
ENSMUSG(	Gjc3	-2.77526	1.06E-06	9.61E-05
ENSMUSG(	mt-Atp6	-2.77224	2.96E-06	0.000232
ENSMUSG(	Serpinh1	-2.76269	0.000862	0.02457
ENSMUSG(	Cpne3	-2.76247	6.45E-07	6.40E-05
ENSMUSG(	Caskin2	-2.75847	5.24E-07	5.39E-05
ENSMUSG(	Qk	-2.75308	1.40E-11	6.18E-09
ENSMUSG(	Cd9	-2.74972	1.32E-07	1.74E-05
ENSMUSG(	P2ry12	-2.74944	3.13E-06	0.000244
ENSMUSG(	Scd1	-2.74831	3.79E-09	7.97E-07
ENSMUSG(	Tcn2	-2.74759	3.13E-05	0.001711
ENSMUSG(	Mfsd2a	-2.74666	1.36E-06	0.000118
ENSMUSG(	Acot11	-2.74472	5.14E-08	7.82E-06
ENSMUSG(	Nrarp	-2.73948	3.91E-07	4.27E-05
ENSMUSG(	Nkx2-9	-2.73943	0.00045	0.01468
ENSMUSG(	Clic4	-2.73778	1.96E-10	6.24E-08
ENSMUSG(	Myoc	-2.73313	2.68E-05	0.001507
ENSMUSG(	Cyfip1	-2.73157	3.08E-07	3.53E-05

ENSMUSG(	Acadl	-2.73051	1.60E-09	3.72E-07
ENSMUSG(	Ptgds	-2.72838	2.70E-06	0.000214
ENSMUSG(	Col9a3	-2.7273	4.48E-05	0.002281
ENSMUSG(	Myrf	-2.72379	2.44E-06	0.000195
ENSMUSG(	Daam2	-2.71915	5.89E-06	0.000421
ENSMUSG(	Bmp7	-2.71873	0.000285	0.010274
ENSMUSG(	Nde1	-2.71358	1.09E-08	2.07E-06
ENSMUSG(	Cbs	-2.71323	3.11E-10	9.33E-08
ENSMUSG(	Arhgef19	-2.71164	2.90E-07	3.39E-05
ENSMUSG(	Vamp8	-2.71148	0.000112	0.004864
ENSMUSG(	Pla2g7	-2.71068	3.78E-09	7.97E-07
ENSMUSG(	Cnp	-2.6954	2.46E-09	5.53E-07
ENSMUSG(	Kif13b	-2.69528	4.25E-08	6.69E-06
ENSMUSG(	Plce1	-2.69459	1.85E-06	0.000155
ENSMUSG(	Sall1	-2.69352	2.90E-05	0.001606
ENSMUSG(	Serping1	-2.68575	9.31E-06	0.000608
ENSMUSG(	Tmem88b	-2.6857	7.53E-09	1.51E-06
ENSMUSG(	Phactr4	-2.6822	0.00116	0.03058
ENSMUSG(	Gjb6	-2.67571	1.90E-08	3.30E-06
ENSMUSG(	Olfml1	-2.67397	2.21E-05	0.001265
ENSMUSG(	Cyp2d22	-2.6667	2.95E-07	3.44E-05
ENSMUSG(	Etv4	-2.66667	0.000112	0.004871
ENSMUSG(	Pgghg	-2.66533	0.000109	0.004759
ENSMUSG(	Gjc2	-2.66456	6.39E-06	0.000449
ENSMUSG(	Cryab	-2.65751	4.18E-11	1.72E-08
ENSMUSG(	Capg	-2.65333	0.000153	0.006322
ENSMUSG(	Trf	-2.65274	1.18E-06	0.000105
ENSMUSG(	Zfp36l2	-2.65022	6.40E-05	0.003085
ENSMUSG(	Tlcd1	-2.64693	6.04E-06	0.000429
ENSMUSG(	Pxmp2	-2.64434	7.52E-05	0.003494
ENSMUSG(	Tspan15	-2.64427	2.24E-06	0.000182
ENSMUSG(	Cfh	-2.64251	0.001058	0.028583
ENSMUSG(	Gatm	-2.63582	8.11E-11	2.96E-08
ENSMUSG(	Stard13	-2.63568	3.40E-06	0.000258
ENSMUSG(	Efemp1	-2.63169	3.09E-05	0.001693
ENSMUSG(	Apln	-2.62914	8.50E-07	8.15E-05
ENSMUSG(	Skor1	-2.62905	0.000599	0.018231
ENSMUSG(	Eva1a	-2.62627	1.43E-07	1.87E-05
ENSMUSG(	Cyp26b1	-2.6188	0.000337	0.011711
ENSMUSG(	Gm15163	-2.61644	5.70E-05	0.002804
ENSMUSG(	Car8	-2.61296	1.54E-10	5.10E-08
ENSMUSG(	Cdc42ep1	-2.60886	1.89E-07	2.37E-05
ENSMUSG(	Slc15a2	-2.59346	1.41E-05	0.000869
ENSMUSG(	Trim25	-2.59086	4.81E-05	0.002419
ENSMUSG(	Abhd3	-2.59013	2.60E-09	5.77E-07
ENSMUSG(	C4b	-2.58859	1.77E-06	0.000149
ENSMUSG(	Selenbp1	-2.58706	2.11E-07	2.57E-05

ENSMUSG(	Nkx6-2	-2.58669	1.72E-08	3.09E-06
ENSMUSG(	Dnaic1	-2.58479	0.00058	0.017748
ENSMUSG(	Ssfa2	-2.58459	3.69E-07	4.10E-05
ENSMUSG(	Kat2b	-2.57887	9.84E-07	9.11E-05
ENSMUSG(	Pbxip1	-2.57438	1.44E-10	4.83E-08
ENSMUSG(	Zc3hav1	-2.57306	2.51E-06	0.0002
ENSMUSG(	Trem2	-2.57238	6.38E-05	0.003084
ENSMUSG(	Nfia	-2.56818	5.09E-07	5.28E-05
ENSMUSG(	Plpp3	-2.56745	3.73E-07	4.12E-05
ENSMUSG(	Sgk1	-2.56377	1.74E-09	4.00E-07
ENSMUSG(	Pllp	-2.56177	3.16E-06	0.000245
ENSMUSG(	Prkd1	-2.56075	0.000234	0.008796
ENSMUSG(	Eya1	-2.55961	5.66E-06	0.000407
ENSMUSG(	Gm42756	-2.55406	0.000689	0.020543
ENSMUSG(	Rab31	-2.55343	1.17E-10	4.00E-08
ENSMUSG(	Tnfaip8	-2.55094	2.08E-05	0.001208
ENSMUSG(	Sash1	-2.55083	5.44E-07	5.55E-05
ENSMUSG(	Inpp5d	-2.54966	0.000676	0.020223
ENSMUSG(	Lamb2	-2.54924	1.99E-06	0.000164
ENSMUSG(	Lpcat2	-2.54889	1.08E-06	9.73E-05
ENSMUSG(	Lims2	-2.54882	8.75E-07	8.32E-05
ENSMUSG(	Eps8l2	-2.54399	0.000109	0.004765
ENSMUSG(	Npepl1	-2.54273	6.74E-05	0.003201
ENSMUSG(	Ppp1r3c	-2.54192	1.57E-07	1.99E-05
ENSMUSG(	Ctsc	-2.54103	4.84E-05	0.002426
ENSMUSG(	Abhd4	-2.54062	1.81E-07	2.28E-05
ENSMUSG(	Lrig1	-2.53701	4.31E-08	6.76E-06
ENSMUSG(	Nfix	-2.53495	2.43E-07	2.89E-05
ENSMUSG(	Arhgap31	-2.53469	2.02E-06	0.000166
ENSMUSG(	Mt2	-2.53462	9.07E-06	0.000594
ENSMUSG(	Tmc6	-2.5324	0.000713	0.021067
ENSMUSG(	Zfp36l1	-2.53181	6.51E-05	0.003124
ENSMUSG(	Lhfp	-2.53024	2.72E-05	0.001527
ENSMUSG(	Rbl1	-2.5239	0.001418	0.035331
ENSMUSG(	Sapcd2	-2.52342	0.000726	0.021354
ENSMUSG(	Cnn3	-2.52319	6.25E-10	1.69E-07
ENSMUSG(	AI464131	-2.52283	2.13E-05	0.00123
ENSMUSG(	Dab2	-2.52084	5.44E-05	0.002683
ENSMUSG(	Gltp	-2.51701	1.30E-08	2.38E-06
ENSMUSG(	Jph1	-2.51681	2.07E-05	0.001208
ENSMUSG(	Insc	-2.51608	3.80E-05	0.002004
ENSMUSG(	Akna	-2.50754	0.001165	0.030644
ENSMUSG(	Wfdc1	-2.50537	0.000841	0.024129
ENSMUSG(	Ccdc122	-2.50319	0.000559	0.01726
ENSMUSG(	Boc	-2.50224	0.001786	0.041344
ENSMUSG(	Elovl1	-2.50197	0.0002	0.007854
ENSMUSG(	Gm28035	-2.4934	0.00076	0.022186

ENSMUSG(	Timp3	-2.49206	1.33E-06	0.000116
ENSMUSG(	Mpst	-2.49049	3.40E-05	0.001838
ENSMUSG(	Pnpla7	-2.48966	6.87E-06	0.000476
ENSMUSG(	Rgma	-2.48955	0.00143	0.035466
ENSMUSG(	Cd82	-2.48649	5.55E-07	5.62E-05
ENSMUSG(	Gab1	-2.48408	1.07E-06	9.70E-05
ENSMUSG(	Mertk	-2.48339	4.03E-05	0.0021
ENSMUSG(	Myo6	-2.48181	2.73E-08	4.47E-06
ENSMUSG(	Evi2a	-2.47724	5.38E-05	0.002659
ENSMUSG(	Thbs2	-2.47338	0.001584	0.038154
ENSMUSG(	Glns-ps1	-2.47047	0.000172	0.006925
ENSMUSG(	Tril	-2.46677	4.86E-08	7.54E-06
ENSMUSG(	Prr5l	-2.4644	2.10E-07	2.57E-05
ENSMUSG(	F3	-2.46093	1.45E-07	1.87E-05
ENSMUSG(	Dcn	-2.45675	9.77E-05	0.004311
ENSMUSG(	Fxyd1	-2.45425	7.28E-05	0.003409
ENSMUSG(	Rasgrp3	-2.45211	0.001021	0.02784
ENSMUSG(	Sorbs3	-2.44915	5.80E-08	8.55E-06
ENSMUSG(	Mitf	-2.44739	0.000302	0.010766
ENSMUSG(	Rcsd1	-2.44275	0.000121	0.005196
ENSMUSG(	D7Ertd443	-2.43869	0.000692	0.020636
ENSMUSG(	A230001M	-2.43817	0.000365	0.012442
ENSMUSG(	Tmem125	-2.43683	1.89E-06	0.000158
ENSMUSG(	Arhgef26	-2.43471	1.67E-06	0.000142
ENSMUSG(	Mag	-2.43226	1.49E-05	0.000912
ENSMUSG(	Dcx	-2.4281	2.58E-05	0.001456
ENSMUSG(	Acap2	-2.42696	2.21E-05	0.001265
ENSMUSG(	Arpc1b	-2.42347	0.000327	0.011462
ENSMUSG(	Bmpr1b	-2.42059	6.13E-05	0.002992
ENSMUSG(	Myo1d	-2.41973	3.72E-06	0.000281
ENSMUSG(	Mical1	-2.41623	0.000245	0.009119
ENSMUSG(	Gad2	-2.41146	1.70E-09	3.93E-07
ENSMUSG(	Ugt8a	-2.41138	4.25E-05	0.002187
ENSMUSG(	Sgk2	-2.41082	0.000442	0.014483
ENSMUSG(	Vstm4	-2.41057	2.75E-05	0.001535
ENSMUSG(	Rn7s2	-2.40807	0.000285	0.010274
ENSMUSG(	Gpr34	-2.407	0.000184	0.007335
ENSMUSG(	Igsf1	-2.40578	7.02E-06	0.000484
ENSMUSG(	Gm26694	-2.40223	0.000116	0.00499
ENSMUSG(	Nemp2	-2.40134	0.00229	0.049854
ENSMUSG(	Sod3	-2.39907	4.07E-05	0.002116
ENSMUSG(	Tjp2	-2.39886	1.33E-06	0.000116
ENSMUSG(	Slco1c1	-2.39827	0.000182	0.007291
ENSMUSG(	Cldn14	-2.39734	0.000531	0.016585
ENSMUSG(	Cyth4	-2.39705	1.58E-05	0.000959
ENSMUSG(	Igfbp7	-2.39524	0.000165	0.00671
ENSMUSG(	Tmod1	-2.39502	1.89E-05	0.001116

ENSMUSG(	Anxa3	-2.39359	8.47E-05	0.003861
ENSMUSG(	Ednrb	-2.38874	1.12E-07	1.51E-05
ENSMUSG(	Scara3	-2.38536	0.001067	0.028756
ENSMUSG(	Zfyve21	-2.38263	1.83E-07	2.30E-05
ENSMUSG(	Emp2	-2.38103	4.78E-06	0.00035
ENSMUSG(	Tnni1	-2.37856	0.000234	0.008796
ENSMUSG(	Mt1	-2.37587	7.00E-08	9.96E-06
ENSMUSG(	Elf1	-2.37474	0.000546	0.016959
ENSMUSG(	Sox6	-2.37077	0.000629	0.018992
ENSMUSG(	Zfyve16	-2.37019	0.000213	0.008207
ENSMUSG(	Zbtb16	-2.36574	0.000205	0.007986
ENSMUSG(	Nts	-2.36526	2.40E-06	0.000194
ENSMUSG(	Pltp	-2.36516	4.33E-07	4.66E-05
ENSMUSG(	Cyp27a1	-2.36167	0.001578	0.038088
ENSMUSG(	Hist2h2be	-2.36045	0.000395	0.013296
ENSMUSG(	H2-K1	-2.35721	9.39E-05	0.004199
ENSMUSG(	Nwd1	-2.35667	7.63E-05	0.003529
ENSMUSG(	Hspb1	-2.35593	0.00058	0.017748
ENSMUSG(	Map2	-2.35508	1.20E-06	0.000106
ENSMUSG(	Pla2g4a	-2.35463	0.000221	0.008429
ENSMUSG(	Sec14l2	-2.35421	2.42E-06	0.000195
ENSMUSG(	AW047730	-2.35137	1.39E-05	0.000865
ENSMUSG(	Triobp	-2.346	0.000268	0.009763
ENSMUSG(	Sox1ot	-2.3429	0.00081	0.023358
ENSMUSG(	Slc6a20a	-2.33085	0.001955	0.044424
ENSMUSG(	Afap1l2	-2.32643	1.76E-05	0.001056
ENSMUSG(	Ntsr2	-2.32612	1.96E-08	3.39E-06
ENSMUSG(	Gjb1	-2.31919	4.47E-06	0.000332
ENSMUSG(	Cav1	-2.31872	5.84E-05	0.002866
ENSMUSG(	Slc12a4	-2.31496	6.90E-05	0.003252
ENSMUSG(	Mmp14	-2.3147	4.54E-05	0.00231
ENSMUSG(	Reep3	-2.30012	2.52E-05	0.001427
ENSMUSG(	Islr	-2.29703	0.002234	0.048968
ENSMUSG(	Ldlrap1	-2.29669	0.000661	0.019848
ENSMUSG(	Stxbp3	-2.28975	4.51E-07	4.83E-05
ENSMUSG(	Prox1	-2.28885	0.000207	0.007995
ENSMUSG(	Nfe2l3	-2.28869	0.001493	0.036476
ENSMUSG(	Neu4	-2.28689	3.55E-05	0.0019
ENSMUSG(	Itgb8	-2.28674	2.10E-07	2.57E-05
ENSMUSG(	Enpp2	-2.28566	8.61E-05	0.003905
ENSMUSG(	Rin2	-2.27789	5.74E-07	5.78E-05
ENSMUSG(	Dhrs4	-2.2766	3.28E-05	0.001791
ENSMUSG(	Ltbr	-2.27458	0.000258	0.009519
ENSMUSG(	Prrg1	-2.27456	1.06E-05	0.000679
ENSMUSG(	Stxbp3-ps	-2.27385	9.48E-05	0.004218
ENSMUSG(	mt-Nd5	-2.27354	2.96E-06	0.000232
ENSMUSG(	Jam2	-2.27305	3.46E-07	3.90E-05

ENSMUSG(	Notch2	-2.27292	0.000918	0.025559
ENSMUSG(	Gm28439	-2.27279	4.27E-06	0.00032
ENSMUSG(	Mcc	-2.27152	3.36E-06	0.000255
ENSMUSG(	mt-Nd2	-2.27047	6.88E-06	0.000476
ENSMUSG(	Rasl11a	-2.26787	0.000903	0.025287
ENSMUSG(	Zfp36	-2.26008	0.001391	0.034848
ENSMUSG(	Plxnb3	-2.25863	6.65E-05	0.003171
ENSMUSG(	Trp53bp2	-2.25772	7.74E-05	0.003564
ENSMUSG(	Pde1c	-2.25735	0.000976	0.026843
ENSMUSG(	Glipr2	-2.2557	0.001655	0.039337
ENSMUSG(	Nkain4	-2.25209	1.28E-06	0.000112
ENSMUSG(	Prex1	-2.25176	5.63E-08	8.34E-06
ENSMUSG(	Slc6a9	-2.25166	8.42E-07	8.11E-05
ENSMUSG(	Ctso	-2.24753	0.000414	0.013758
ENSMUSG(	Gstm7	-2.24613	5.10E-08	7.81E-06
ENSMUSG(	Gm973	-2.24445	1.51E-05	0.00092
ENSMUSG(	Mmd2	-2.23672	8.92E-09	1.75E-06
ENSMUSG(	Cyp2j6	-2.23501	0.000311	0.011042
ENSMUSG(	Ifit3	-2.23423	1.32E-05	0.000827
ENSMUSG(	Tmc7	-2.2339	0.000167	0.006773
ENSMUSG(	Icosl	-2.23292	0.001086	0.029216
ENSMUSG(	Id1	-2.22849	0.000349	0.012053
ENSMUSG(	Fbxo32	-2.22405	0.001308	0.033253
ENSMUSG(	Mtmr10	-2.2217	2.84E-05	0.001581
ENSMUSG(	Ptpn13	-2.22066	0.001037	0.028238
ENSMUSG(	Lpp	-2.22043	0.001241	0.031994
ENSMUSG(	Gbp7	-2.21988	0.002207	0.048667
ENSMUSG(	Phldb1	-2.21795	2.48E-07	2.94E-05
ENSMUSG(	Gpm6b	-2.21701	2.51E-08	4.16E-06
ENSMUSG(	Gm45767	-2.21442	9.45E-05	0.004212
ENSMUSG(	Tsc22d4	-2.21159	6.16E-08	8.92E-06
ENSMUSG(	Wls	-2.21145	0.000402	0.013453
ENSMUSG(	mt-Nd4	-2.21069	1.91E-05	0.001126
ENSMUSG(	mt-Nd1	-2.20287	5.18E-05	0.002582
ENSMUSG(	Sspn	-2.19549	0.000117	0.005046
ENSMUSG(	Rn7s1	-2.19384	0.000225	0.00854
ENSMUSG(	Hip1	-2.18253	1.80E-05	0.001077
ENSMUSG(	Prex2	-2.17503	2.44E-05	0.001389
ENSMUSG(	Gm9791	-2.1749	0.001095	0.029389
ENSMUSG(	Pm20d1	-2.16986	0.000323	0.011366
ENSMUSG(	Mbp	-2.16879	1.58E-05	0.000961
ENSMUSG(	Unc93b1	-2.16566	5.04E-05	0.00252
ENSMUSG(	Gm2a	-2.1591	2.17E-08	3.71E-06
ENSMUSG(	Lmx1a	-2.15875	9.71E-07	9.07E-05
ENSMUSG(	Mob3b	-2.15837	3.36E-05	0.001823
ENSMUSG(	Gm2115	-2.15632	7.57E-05	0.003505
ENSMUSG(	Bcan	-2.1556	3.97E-07	4.31E-05

ENSMUSG(	Cpq	-2.1538	3.29E-06	0.000252
ENSMUSG(	Entpd2	-2.15288	0.000254	0.009394
ENSMUSG(	Sugct	-2.14569	3.71E-05	0.001973
ENSMUSG(	Gramd3	-2.14376	6.44E-05	0.003096
ENSMUSG(	Serinc5	-2.14148	1.87E-05	0.001108
ENSMUSG(	Frmd8	-2.13891	1.00E-07	1.37E-05
ENSMUSG(	Gpr37	-2.13599	0.000188	0.007448
ENSMUSG(	Inf2	-2.13349	0.000499	0.015825
ENSMUSG(	Kansl1l	-2.13326	0.000783	0.022773
ENSMUSG(	Myo9b	-2.13095	9.66E-05	0.004278
ENSMUSG(	Lrig3	-2.12932	0.001781	0.041304
ENSMUSG(	Rassf2	-2.12331	1.01E-06	9.26E-05
ENSMUSG(	Ly6a	-2.12213	0.001704	0.040136
ENSMUSG(	mt-Nd6	-2.12064	4.20E-05	0.002168
ENSMUSG(	Oplah	-2.11967	3.35E-05	0.001822
ENSMUSG(	Susd5	-2.11802	0.000513	0.016127
ENSMUSG(	Tfap2b	-2.11252	3.33E-06	0.000253
ENSMUSG(	Tjp1	-2.1087	1.92E-05	0.001126
ENSMUSG(	Tac1	-2.09784	1.31E-05	0.000825
ENSMUSG(	Mdc1	-2.09621	0.000445	0.014571
ENSMUSG(	Lactb2	-2.09599	2.91E-05	0.001606
ENSMUSG(	Rorb	-2.09415	5.37E-07	5.50E-05
ENSMUSG(	Abcd2	-2.09158	2.17E-06	0.000177
ENSMUSG(	Rnf135	-2.08324	0.001111	0.0296
ENSMUSG(	Rhoc	-2.07828	0.001783	0.041304
ENSMUSG(	Anxa4	-2.07754	0.000362	0.012386
ENSMUSG(	Fryl	-2.0767	0.000319	0.011261
ENSMUSG(	Chil1	-2.07627	1.77E-05	0.001061
ENSMUSG(	Creb5	-2.07234	0.001409	0.035132
ENSMUSG(	mt-Atp8	-2.07109	0.000463	0.015007
ENSMUSG(	Trim59	-2.06265	1.87E-05	0.001107
ENSMUSG(	Cnn2	-2.05847	0.000221	0.008422
ENSMUSG(	Arhgdib	-2.05061	0.00014	0.005886
ENSMUSG(	Fermt2	-2.04229	3.53E-07	3.96E-05
ENSMUSG(	mt-Cytb	-2.04052	0.000154	0.006372
ENSMUSG(	Gpt2	-2.03673	1.66E-06	0.000142
ENSMUSG(	Csnk1g1	-2.03017	0.001371	0.034505
ENSMUSG(	Acaa2	-2.02819	9.37E-06	0.00061
ENSMUSG(	D16Ertd47:	-2.02775	0.000436	0.014334
ENSMUSG(	Ttyh1	-2.02475	5.55E-08	8.26E-06
ENSMUSG(	Slc7a2	-2.02414	0.000461	0.014967
ENSMUSG(	St18	-2.02244	0.000127	0.005397
ENSMUSG(	Irak2	-2.01924	3.07E-06	0.00024
ENSMUSG(	Sh3bgr	-2.01834	0.000425	0.014074
ENSMUSG(	Fam53b	-2.01425	3.39E-05	0.001838
ENSMUSG(	Smtnl2	-2.01268	0.000225	0.00854
ENSMUSG(	Gm10925	-2.01209	0.000155	0.006389

ENSMUSG(	Ddo	-2.00459	8.27E-06	0.000549
ENSMUSG(	Gm35040	-2.00175	0.001707	0.040136
ENSMUSG(	Gpr146	-2.0002	0.000121	0.005173
ENSMUSG(	Cdr1	-1.99861	1.10E-06	9.90E-05
ENSMUSG(	Rhpn2	-1.99463	1.01E-05	0.000652
ENSMUSG(	Ino80d	-1.98951	0.00071	0.021042
ENSMUSG(	Myo10	-1.98932	5.36E-05	0.002652
ENSMUSG(	Ppp1r18	-1.98597	0.000668	0.020027
ENSMUSG(	Aldh6a1	-1.98148	1.08E-06	9.73E-05
ENSMUSG(	Paqr8	-1.98008	2.72E-05	0.001528
ENSMUSG(	Cmtm6	-1.97941	9.53E-05	0.004228
ENSMUSG(	Appl2	-1.97883	9.83E-07	9.11E-05
ENSMUSG(	Hsd3b7	-1.97679	0.000279	0.010103
ENSMUSG(	Zeb1	-1.97504	7.97E-05	0.003655
ENSMUSG(	Bcas1	-1.96609	0.00048	0.015381
ENSMUSG(	Trps1	-1.96434	0.000372	0.012684
ENSMUSG(	Hes5	-1.96214	4.32E-05	0.002212
ENSMUSG(	Ss18	-1.96149	2.67E-05	0.001507
ENSMUSG(	Serhl	-1.9595	0.000554	0.017138
ENSMUSG(	Stk17b	-1.95939	0.000491	0.015612
ENSMUSG(	Mobp	-1.95913	3.89E-05	0.002034
ENSMUSG(	Emilin2	-1.9537	0.001951	0.044396
ENSMUSG(	Gna13	-1.94586	3.73E-05	0.001973
ENSMUSG(	Gpam	-1.94438	1.09E-05	0.000698
ENSMUSG(	Nfib	-1.93753	0.000155	0.006395
ENSMUSG(	Aldoc	-1.93134	2.05E-07	2.53E-05
ENSMUSG(	Apc	-1.93086	2.00E-06	0.000165
ENSMUSG(	Sft2d2	-1.93048	9.18E-05	0.004119
ENSMUSG(	Slc12a2	-1.92772	0.000114	0.004927
ENSMUSG(	Dpyd	-1.92738	9.13E-05	0.004105
ENSMUSG(	Exoc3l4	-1.92681	0.000161	0.006542
ENSMUSG(	Arhgap17	-1.92566	0.000328	0.011479
ENSMUSG(	Il1rap	-1.92006	0.001054	0.028499
ENSMUSG(	Tmem229a	-1.91544	1.52E-06	0.000131
ENSMUSG(	Ifi27	-1.91461	1.95E-06	0.000162
ENSMUSG(	Snhg14	-1.91168	0.001148	0.030445
ENSMUSG(	Tnpo1	-1.90835	3.26E-06	0.00025
ENSMUSG(	Fads2	-1.9073	4.84E-05	0.002426
ENSMUSG(	Dbi	-1.90362	1.14E-05	0.000723
ENSMUSG(	Tspan14	-1.90263	0.000346	0.011973
ENSMUSG(	Psat1	-1.90097	2.12E-07	2.57E-05
ENSMUSG(	Myo1b	-1.89921	0.001114	0.029647
ENSMUSG(	Acad11	-1.89714	5.28E-05	0.002625
ENSMUSG(	St6galnac3	-1.89434	0.001687	0.039884
ENSMUSG(	Prelp	-1.88776	7.41E-05	0.003455
ENSMUSG(	Fgfr2	-1.88737	0.000263	0.009642
ENSMUSG(	Serpine2	-1.88612	1.30E-05	0.000817

ENSMUSG(	Slc25a13	-1.88443	0.000329	0.011479
ENSMUSG(	Npas3	-1.88434	3.71E-05	0.001973
ENSMUSG(	Skap2	-1.88236	8.27E-06	0.000549
ENSMUSG(	Itgav	-1.88166	0.001089	0.029273
ENSMUSG(	Lpcat3	-1.88086	0.000719	0.021228
ENSMUSG(	Gpld1	-1.87788	0.00029	0.010429
ENSMUSG(	Abtb2	-1.87612	0.000104	0.004571
ENSMUSG(	Fads1	-1.87557	2.90E-05	0.001606
ENSMUSG(	Slc13a3	-1.87271	0.000473	0.015245
ENSMUSG(	Mvp	-1.87078	3.72E-05	0.001973
ENSMUSG(	Scd2	-1.8698	7.91E-05	0.003633
ENSMUSG(	Gm28437	-1.86905	0.00057	0.017488
ENSMUSG(	Spg11	-1.86694	0.000143	0.00597
ENSMUSG(	Fam163a	-1.86693	0.001156	0.030514
ENSMUSG(	mt-Co3	-1.86622	0.000702	0.020855
ENSMUSG(	Hmox1	-1.86558	0.000389	0.013141
ENSMUSG(	Fam181b	-1.86	7.65E-06	0.000517
ENSMUSG(	Spsb1	-1.85791	0.000188	0.007448
ENSMUSG(	Mboat2	-1.84462	5.73E-06	0.00041
ENSMUSG(	Fbxo30	-1.83727	0.000452	0.014703
ENSMUSG(	Zeb2	-1.83636	0.000842	0.024137
ENSMUSG(	Gm28661	-1.82733	0.001278	0.032668
ENSMUSG(	Pacsin3	-1.8247	3.80E-05	0.002004
ENSMUSG(	Elovl2	-1.82437	0.001268	0.032557
ENSMUSG(	Tanc1	-1.81822	6.14E-05	0.002992
ENSMUSG(	Necap2	-1.81779	1.60E-05	0.00097
ENSMUSG(	Arap2	-1.7968	0.000604	0.018353
ENSMUSG(	Tgfa	-1.79617	3.89E-05	0.002033
ENSMUSG(	mt-Co2	-1.78964	0.001394	0.034878
ENSMUSG(	4930402H2	-1.78776	1.55E-05	0.000942
ENSMUSG(	Plekhb1	-1.7872	0.000178	0.007159
ENSMUSG(	Phka1	-1.7845	0.000156	0.006396
ENSMUSG(	Lrrc1	-1.783	0.000631	0.019035
ENSMUSG(	2-Sep	-1.78107	2.60E-06	0.000206
ENSMUSG(	Hr	-1.77898	0.000724	0.021311
ENSMUSG(	Fmo5	-1.77791	0.001833	0.042248
ENSMUSG(	Crhbp	-1.77701	0.000533	0.016614
ENSMUSG(	Trp53bp1	-1.77644	0.000213	0.008195
ENSMUSG(	mt-Co1	-1.77558	0.001194	0.031172
ENSMUSG(	Id2	-1.77442	4.52E-06	0.000333
ENSMUSG(	Syne1	-1.76518	1.95E-05	0.001145
ENSMUSG(	Llgl1	-1.7627	8.06E-06	0.000541
ENSMUSG(	Cxcl14	-1.76069	5.91E-05	0.002897
ENSMUSG(	Sqor	-1.76011	0.000184	0.007335
ENSMUSG(	Prdx6	-1.75966	7.56E-06	0.000514
ENSMUSG(	Cdk19	-1.75142	0.001208	0.031487
ENSMUSG(	Rest	-1.74844	0.001403	0.035032

ENSMUSG(	4-Sep	-1.74122	9.32E-07	8.75E-05
ENSMUSG(	Nfat5	-1.73337	0.000469	0.015146
ENSMUSG(	Prkcq	-1.72801	0.000295	0.010568
ENSMUSG(	Rida	-1.72441	4.58E-05	0.002323
ENSMUSG(	Cald1	-1.71777	0.000709	0.021015
ENSMUSG(	Cavin1	-1.71471	0.001522	0.036916
ENSMUSG(	Mettl7a1	-1.71459	0.000396	0.013303
ENSMUSG(	Abhd5	-1.7108	0.000221	0.008422
ENSMUSG(	Cep97	-1.70279	0.000499	0.015828
ENSMUSG(	Peli2	-1.69452	0.000331	0.011534
ENSMUSG(	Il18	-1.69404	0.00019	0.007515
ENSMUSG(	Spata13	-1.69008	0.001881	0.04321
ENSMUSG(	2810468N(	-1.67917	7.35E-05	0.003438
ENSMUSG(	Nek9	-1.67092	0.000195	0.007679
ENSMUSG(	Fam234a	-1.66335	0.000267	0.009757
ENSMUSG(	Fut10	-1.66047	0.002088	0.046501
ENSMUSG(	Usp53	-1.65664	0.00027	0.009827
ENSMUSG(	Fah	-1.65066	2.91E-05	0.001606
ENSMUSG(	Smpdl3a	-1.64953	0.001873	0.043065
ENSMUSG(	Tns1	-1.64832	8.62E-05	0.003905
ENSMUSG(	Msn	-1.64691	0.001152	0.030514
ENSMUSG(	Carmil1	-1.64587	0.000147	0.006107
ENSMUSG(	Dmd	-1.64397	0.000272	0.009882
ENSMUSG(	Slc27a1	-1.6302	0.000186	0.007397
ENSMUSG(	Tpmt	-1.62933	0.000934	0.025896
ENSMUSG(	Hacl1	-1.62728	0.001697	0.040042
ENSMUSG(	Ppfibp2	-1.62059	0.001824	0.042138
ENSMUSG(	Yes1	-1.61638	0.001206	0.031445
ENSMUSG(	Snx22	-1.61319	0.000552	0.017117
ENSMUSG(	Klf3	-1.60857	0.00051	0.016081
ENSMUSG(	Vamp3	-1.60125	0.000377	0.012833
ENSMUSG(	Phlpp1	-1.5998	8.61E-05	0.003905
ENSMUSG(	Kdm3b	-1.59643	0.00048	0.015381
ENSMUSG(	Add3	-1.59226	2.18E-05	0.001253
ENSMUSG(	Neat1	-1.59022	0.000939	0.026024
ENSMUSG(	Nbeal1	-1.58895	0.00066	0.019848
ENSMUSG(	Pir	-1.58627	0.001044	0.028328
ENSMUSG(	Maob	-1.58562	3.84E-05	0.002015
ENSMUSG(	Fkbp9	-1.58474	0.002224	0.048847
ENSMUSG(	Plekhd1	-1.58036	0.000883	0.024922
ENSMUSG(	Klf15	-1.5782	6.97E-05	0.003277
ENSMUSG(	Sbf2	-1.57742	0.000199	0.007816
ENSMUSG(	Etv5	-1.5625	0.000232	0.008759
ENSMUSG(	Acsl6	-1.56173	0.000206	0.007986
ENSMUSG(	Eci1	-1.55667	0.000433	0.014284
ENSMUSG(	Ccnd3	-1.55472	0.000544	0.016929
ENSMUSG(	Kctd5	-1.53958	0.00014	0.005886

ENSMUSG(	Ctnnd1	-1.53906	0.000547	0.016987
ENSMUSG(	Cat	-1.53286	4.80E-05	0.002419
ENSMUSG(	Ndrg1	-1.53231	2.52E-05	0.001427
ENSMUSG(	Kmt2c	-1.52988	0.002004	0.045071
ENSMUSG(	Ankhd1	-1.52629	0.000396	0.013303
ENSMUSG(	Plcd4	-1.52499	0.002049	0.045726
ENSMUSG(	Lims1	-1.51847	0.000877	0.024851
ENSMUSG(	Nup160	-1.51511	0.001981	0.0448
ENSMUSG(	Trim36	-1.50997	0.000762	0.022215
ENSMUSG(	Hacd2	-1.50793	0.000787	0.022821
ENSMUSG(	Asrgl1	-1.50534	0.000257	0.009505
ENSMUSG(	Ccnd1	-1.49758	0.001161	0.03058
ENSMUSG(	Wnk1	-1.49475	0.00035	0.012068
ENSMUSG(	Thrsp	-1.48916	0.001518	0.036916
ENSMUSG(	Nr3c2	-1.48847	0.001482	0.03633
ENSMUSG(	Ephx2	-1.48775	0.001007	0.027552
ENSMUSG(	Dnm2	-1.48546	9.07E-05	0.004094
ENSMUSG(	Esr1	-1.48367	0.001186	0.031059
ENSMUSG(	Zcchc24	-1.48109	0.000205	0.007986
ENSMUSG(	Wasf2	-1.47372	0.002166	0.047964
ENSMUSG(	Sec14l1	-1.46735	0.000424	0.014053
ENSMUSG(	Birc6	-1.46246	0.000287	0.010347
ENSMUSG(	Pcca	-1.46141	0.00137	0.034505
ENSMUSG(	Golgb1	-1.45854	0.002242	0.049108
ENSMUSG(	Tcf12	-1.45703	0.000562	0.017302
ENSMUSG(	Mob1a	-1.44404	0.002265	0.049444
ENSMUSG(	Lap3	-1.43988	0.000239	0.008963
ENSMUSG(	B2m	-1.43826	0.000472	0.015228
ENSMUSG(	Nufip2	-1.435	0.000749	0.021952
ENSMUSG(	Ankrd52	-1.42635	0.001693	0.039976
ENSMUSG(	Mcur1	-1.42559	0.000979	0.02689
ENSMUSG(	Smg1	-1.42432	0.001334	0.033815
ENSMUSG(	Abl1	-1.42176	0.001953	0.044418
ENSMUSG(	Igfbp4	-1.42035	0.001658	0.039368
ENSMUSG(	Gabpa	-1.41672	0.00222	0.048804
ENSMUSG(	Crot	-1.41392	0.000483	0.015433
ENSMUSG(	Otud7b	-1.41365	0.000326	0.011433
ENSMUSG(	Ivd	-1.41287	0.000662	0.019853
ENSMUSG(	Klhl4	-1.41254	0.001631	0.039082
ENSMUSG(	Tcf4	-1.41207	0.000619	0.018714
ENSMUSG(	Htt	-1.41166	0.000789	0.02286
ENSMUSG(	Dbndd2	-1.41071	0.000554	0.017138
ENSMUSG(	Luzp2	-1.40829	0.00036	0.012332
ENSMUSG(	Hexb	-1.40389	0.001163	0.030604
ENSMUSG(	C130021I2(	-1.40012	0.001274	0.0326
ENSMUSG(	Cplx2	-1.39123	0.000241	0.008976
ENSMUSG(	Pex5l	-1.39055	0.000724	0.021311

ENSMUSG(	Ptch1	-1.38995	0.001155	0.030514
ENSMUSG(	Zyx	-1.38553	0.001284	0.032721
ENSMUSG(	Pnpla2	-1.38542	0.000476	0.015301
ENSMUSG(	Hipk2	-1.38316	0.000507	0.016029
ENSMUSG(	Vwa8	-1.37813	0.001626	0.039022
ENSMUSG(	Kmt2d	-1.3754	0.000463	0.015
ENSMUSG(	Tmcc3	-1.37391	0.000516	0.016184
ENSMUSG(	Pcx	-1.37292	0.000871	0.024738
ENSMUSG(	Agl	-1.36713	0.001093	0.029367
ENSMUSG(	Aldh4a1	-1.35059	0.001216	0.031617
ENSMUSG(	Gm13910	-1.33801	0.000785	0.0228
ENSMUSG(	Nacc2	-1.3339	0.000433	0.014284
ENSMUSG(	Chm	-1.33127	0.001479	0.036288
ENSMUSG(	Znfx1	-1.3277	0.001587	0.038198
ENSMUSG(	Dip2a	-1.32055	0.002265	0.049444
ENSMUSG(	Hsd17b4	-1.31816	0.00075	0.021952
ENSMUSG(	Cpped1	-1.31036	0.000907	0.025313
ENSMUSG(	Hadha	-1.30279	0.000683	0.0204
ENSMUSG(	Ppp1r16b	-1.29506	0.000569	0.017482
ENSMUSG(	Ipo7	-1.29299	0.001407	0.035106
ENSMUSG(	Limch1	-1.27815	0.001054	0.028499
ENSMUSG(	Pik3r1	-1.26723	0.001758	0.040839
ENSMUSG(	Rora	-1.26328	0.000853	0.024333
ENSMUSG(	Pnn	-1.25372	0.00158	0.038125
ENSMUSG(	Dazap2	-1.24899	0.001189	0.0311
ENSMUSG(	Nacad	-1.24881	0.000886	0.024922
ENSMUSG(	Anxa5	-1.22807	0.000561	0.017297
ENSMUSG(	Ak3	-1.21985	0.001982	0.0448
ENSMUSG(	Washc2	-1.21324	0.001108	0.029563
ENSMUSG(	Hcfc1	-1.21195	0.001214	0.031593
ENSMUSG(	Scp2	-1.20475	0.001713	0.040146
ENSMUSG(	Tmem47	-1.19935	0.001281	0.0327
ENSMUSG(	Nr3c1	-1.19452	0.001672	0.039571
ENSMUSG(	Capn2	-1.1902	0.001499	0.036598
ENSMUSG(	Adk	-1.18818	0.001796	0.041551
ENSMUSG(	Eps15	-1.18777	0.001522	0.036916
ENSMUSG(	Hook3	-1.18322	0.001919	0.043773
ENSMUSG(	Dtna	-1.18206	0.002021	0.045258
ENSMUSG(	Jup	-1.18194	0.001508	0.036777
ENSMUSG(	Sirt2	-1.14947	0.001893	0.043398
ENSMUSG(	Fnbp1	-1.14011	0.001987	0.044849

Supplementary Table 2. Summary of statistical analyses

Figure	Sample size (n)	Statistical test	P values
1i	Mice: 3<br>Brain slices: 20	Two-tailed unpaired t-test	t=5.056, df=38<br>*P<0.0001*
2e	Warming: 14 mice<br>Cooling: 14 mice	Two-tailed paired t test	t=3.398, df=13, *P=0.0048*
2h	GCaMP6: 10 mice<br>21°C: 30 trials<br>16°C: 34 trials<br>10°C: 32 trials<br>4°C: 33 trials	Brown-Forsythe and Welch ANOVA tests<br>Don't correct for multiple comparisons test	*F* (DFn, DFd) 0.4612 (3.000, 122.7), P=0.7099*<br>Multiple comparisons:<br>21°C vs. 16°C,*P=0.4077*<br>21°C vs. 10°C, *P=0.3677*<br>21°C vs. 4°C, *P=0.2756*<br>16°C vs. 10°C, *P=0.9587*<br>16°C vs. 4°C, *P=0.7521*<br>10°C vs. 4°C, *P=0.7838*
2i	GCaMP6: 10 mice<br>21°C: 30 trials<br>16°C: 34 trials<br>10°C: 32 trials<br>4°C: 33 trials	Brown-Forsythe and Welch ANOVA tests<br>Don't correct for multiple comparisons test	*F* (DFn, DFd) 17.70 (3.000, 116.8), P<0.0001*<br>Multiple comparisons:<br>21°C vs. 16°C,*P=0.2288*<br>21°C vs. 10°C, *P<0.0001*<br>21°C vs. 4°C, *P<0.0001*<br>16°C vs. 10°C, *P=0.0021*<br>16°C vs. 4°C, *P<0.0001*<br>10°C vs. 4°C, *P=0.0547*
2k	GCaMP6: 10 mice<br>35-20°C: 20 trials<br>32-17°C: 17 trials<br>25-10°C: 26 trials<br>29-14°C: 17 trials	Brown-Forsythe and Welch ANOVA tests<br>Don't correct for multiple comparisons test	*F* (DFn, DFd) 0.1478 (3.000, 66.04), P=0.9308*<br>*Multiple comparisons:*<br>35-20°C vs. 32-17°C, *P=0.6186*<br>35-20°C vs. 25-10°C, *P=0.4975*<br>35-20°C vs. 29-14°C, *P=0.9098*<br>32-17°C vs. 25-10°C, *P=0.8965*<br>32-17°C vs. 29-14°C, *P=0.7182*<br>25-10°C vs. 29-14°C, *P=0.6058*
2l	GCaMP6: 10 mice<br>35-20°C: 20 trials<br>32-17°C: 17 trials<br>25-10°C: 26 trials<br>29-14°C: 17 trials	Brown-Forsythe and Welch ANOVA tests<br>Don't correct for multiple comparisons test	*F* (DFn, DFd) 25.48 (3.000, 103.9), P=0.1478*<br>*Multiple comparisons:*<br>35-20°C vs. 32-17°C, *P=0.0484*<br>35-20°C vs. 25-10°C, *P<0.0001*<br>35-20°C vs. 29-14°C, *P<0.0001*<br>32-17°C vs. 25-10°C, *P<0.0001*<br>32-17°C vs. 29-14°C, *P=0.0084*<br>25-10°C vs. 29-14°C, *P=0.0033*
2n	GCaMP6: 10 mice<br>0.1°C/s:19 trials<br>0.2°C/s: 20 trials<br>0.4°C/s: 20 trials<br>0.7°C/s: 20 trials	Brown-Forsythe and Welch ANOVA tests<br>Don't correct for multiple comparisons test	*F* (DFn, DFd) 1.693 (3.000, 57.55), P=0.1785*<br>*Multiple comparisons:*<br>0.1°C/s vs. 0.2°C/s, *P=0.0356*<br>0.1°C/s vs. 0.4°C/s, *P=0.0696*<br>0.1°C/s vs. 0.7°C/s, *P=0.0853*<br>0.2°C/s vs. 0.4°C/s, *P=0.7986*<br>0.2°C/s vs. 0.7°C/s, *P=0.4623*<br>0.4°C/s vs. 0.7°C/s, *P=0.6562*
2o	GCaMP6: 10 mice<br>0.1°C/s:19 trials<br>0.2°C/s: 20 trials<br>0.4°C/s: 20 trials<br>0.7°C/s: 20 trials	Brown-Forsythe and Welch ANOVA tests<br>Don't correct for multiple comparisons test	*F* (DFn, DFd) 0.4130 (3.000, 71.93), P=0.7442*<br>*Multiple comparisons:*<br>0.1°C/s vs. 0.2°C/s, *P=0.8573*<br>0.1°C/s vs. 0.4°C/s, *P=0.3253*<br>0.1°C/s vs. 0.7°C/s, *P=0.6648*<br>0.2°C/s vs. 0.4°C/s, *P=0.4287*<br>0.2°C/s vs. 0.7°C/s, *P=0.8226*<br>0.4°C/s vs. 0.7°C/s, *P=0.5019*
2s	Warming: 6 mice<br>Cooling: 6 mice	Two-tailed paired t test	t=5.560, df=5, *P=0.0026*
2t	Warming: 6 mice<br>Cooling: 6 mice	Two-tailed paired t test	t=4.319, df=5, *P=0.0076*
3c	GFP: 10 mice<br>DMH^{only} projecting LPB<br>TeNT: 9 mice	RM two-way ANOVA<br>factor one: virus (GFP, DMH^{only} projecting LPB TeNT)<br>factor two: time<br>Uncorrected Fisher's LSD test	Virus: *F (1, 17) = 2.664*, *P=0.1210*<br>Time: *F (2.199, 37.38) = 5.516*,*P=0.0065*<br>Interaction: *F (360, 6120) = 2.338*, *P<0.0001*<br>Multiple comparisons:<br>GFP vs. DMH^{only} projecting LPB TeNT:<br>95 min – 108 min, *P<0.0493*<br>117 min – 120 min, *P<0.0488*<br>123 min – 130 min, *P<0.0495*
	DMH^{only} projecting LPB<br>TeNT: 9 mice<br>POA^{only} projecting LPB<br>TeNT: 7mice	RM two-way ANOVA<br>factor one: virus (DMH^{only} projecting LPB TeNT, POA^{only} projecting LPB TeNT)<br>factor two: time<br>Uncorrected Fisher's LSD test	Virus: *F (1, 14) = 0.09332*, *P=0.7645*<br>Time: *F (2.201, 30.82) = 8.153*, *P=0.0011*<br>Interaction: *F (360, 5040) = 0.7286*, *P>0.9999*<br>Multiple comparisons:<br>POA^{only} projecting LPB TeNT vs. DMH^{only} projecting LPB TeNT:<br>*P>0.1575*
3d	GFP: 10 mice<br>DMH^{only} projecting LPB<br>TeNT: 8 mice<br>POA^{only} projecting LPB<br>TeNT: 7mice	RM two-way ANOVA<br>factor one: virus (GFP, DMH^{only} projecting LPB TeNT, POA^{only} projecting LPB TeNT)<br>factor two: time<br>Uncorrected Fisher's LSD test	Virus: *F (2, 22) = 2.757*, *P=0.0854*<br>Time: *F (2.837, 62.40) = 14.82*, *P<0.0001*<br>Interaction: *F (720, 7920) = 1.745*, *P<0.0001*<br>Multiple comparisons:<br>GFP vs. DMH^{only} projecting LPB TeNT:<br>15 min – 74 min, *P<0.0466*<br>220 min – 300 min, *P<0.0193*

(*table continued on the next page*)

Supplementary Table 2. Summary of statistical analyses

Figure	Sample size (n)	Statistical test	P values
3e	GFP: 10 mice<br>DMH^{only} projecting LPB TeNT: 8 mice	RM two-way ANOVA<br>factor one: virus (GFP, DMH^{only} projecting LPB TeNT)<br>factor two: time<br>Uncorrected Fisher's LSD test	Virus: $F(1, 16) = 10.66$, $P=0.0049$<br>Time: $F(360, 5760) = 28.00$, $P<0.0001$<br>Interaction: $F(360, 5760) = 5.486$, $P<0.0001$<br>Multiple comparisons:<br>GFP vs. DMH^{only} projecting LPB TeNT:<br>19 min – 69 min, $P<0.0498$<br>159 min – 300 min, $P<0.0098$
	DMH^{only} projecting LPB TeNT: 8 mice<br>POA^{only} projecting LPB TeNT: 7mice	RM two-way ANOVA<br>factor one: virus (DMH^{only} projecting LPB TeNT, POA^{only} projecting LPB TeNT)<br>factor two: time<br>Uncorrected Fisher's LSD test	Virus: $F(1, 13) = 0.03077$, $P=0.8635$<br>Time: $F(360, 4680) = 36.17$, $P<0.0001$<br>Interaction: $F(360, 4680) = 0.4357$, $P>0.9999$<br>Multiple comparisons:<br>DMH^{only} projecting LPB TeNT vs. POA^{only} projecting LPB TeNT: $P>0.1381$
3f	GFP: 10 mice<br>DMH^{only} projecting LPB TeNT: 8 mice	RM two-way ANOVA<br>factor one: virus (GFP, DMH^{only} projecting LPB TeNT)<br>factor two: time<br>Uncorrected Fisher's LSD test	Virus: $F(1, 16) = 8.921$, $P=0.0087$<br>Time: $F(360, 5760) = 32.63$, $P<0.0001$<br>Interaction: $F(360, 5760) = 5.171$, $P<0.0001$<br>Multiple comparisons:<br>GFP vs. DMH^{only} projecting LPB TeNT: 57 min – 300 min, $P<0.0088$
	DMH^{only} projecting LPB TeNT: 8 mice<br>POA^{only} projecting LPB TeNT: 7mice	RM two-way ANOVA<br>factor one: virus (DMH^{only} projecting LPB TeNT, POA^{only} projecting LPB TeNT)<br>factor two: time<br>Uncorrected Fisher's LSD test	Virus: $F(1, 13) = 2.382$, $P=0.1467$<br>Time: $F(360, 4680) = 47.96$, $P<0.0001$<br>Interaction: $F(360, 4680) = 1.279$, $P=0.0004$<br>Multiple comparisons:<br>DMH^{only} projecting LPB TeNT vs. POA^{only} projecting LPB TeNT:<br>16 min – 62 min, $P<0.0483$
3h	GFP: 10<br>DMH^{LPB} blocking: 10	RM two-way ANOVA<br>factor one: virus (GFP, DMH^{LPB} blocking)<br>factor two: time<br>Uncorrected Fisher's LSD test	Virus: $F(1, 18) = 29.88$, $P<0.0001$<br>Time: $F(4.518, 81.32) = 15.14$, $P<0.0001$<br>Interaction: $F(360, 4680) = 6.670$, $P<0.0001$<br>Multiple comparisons: 37 min – 300 min, $P<0.0075$
	POA^{LPB} blocking: 9<br>Co-blocking: 6	RM two-way ANOVA<br>factor one: virus (POA^{LPB} blocking, Co-blocking)<br>factor two: time<br>Uncorrected Fisher's LSD test	Virus: $F(1, 13) = 3.875$, $P=0.0707$<br>Time: $F(2.126, 27.64) = 24.71$, $P<0.0001$<br>Interaction: $F(360, 4680) = 1.089$, $P=0.1264$<br>Multiple comparisons: 75 min – 149 min, $P<0.0487$
	POA^{LPB} blocking: 9<br>DMH^{LPB} blocking: 10	RM two-way ANOVA<br>factor one: virus (POA^{LPB} blocking, DMH^{LPB} blocking)<br>factor two: time<br>Uncorrected Fisher's LSD test	Virus: $F(1, 17) = 0.9149$, $P=0.3522$<br>Time: $F(2.349, 39.93) = 27.73$, $P<0.0001$<br>Interaction: $F(360, 6120) = 1.761$, $P<0.0001$<br>Multiple comparisons: 35 min – 54 min, $P<0.0453$
3i	GFP: 10<br>DMH^{LPB} blocking: 10	RM two-way ANOVA<br>factor one: virus (GFP, DMH^{LPB} blocking)<br>factor two: time<br>Uncorrected Fisher's LSD test	Virus: $F(1, 18) = 22.52$, $P=0.0002$<br>Time: $F(3.450, 62.11) = 37.24$, $P<0.0001$<br>Interaction: $F(360, 6480) = 11.66$, $P<0.0001$<br>Multiple comparisons: 7 min – 300 min, $P<0.0095$
	POA^{LPB} blocking: 9<br>Co-blocking: 6	RM two-way ANOVA<br>factor one: virus (POA^{LPB} blocking, Co-blocking)<br>factor two: time<br>Uncorrected Fisher's LSD test	Virus: $F(1, 13) = 4.561$, $P=0.0523$<br>Time: $F(2.316, 30.10) = 53.89$, $P<0.0001$<br>Interaction: $F(360, 4680) = 6.098$, $P<0.0001$<br>Multiple comparisons: 116 min – 300 min, $P<0.0495$
	POA^{LPB} blocking: 9<br>DMH^{LPB} blocking: 10	RM two-way ANOVA<br>factor one: virus POA^{LPB} blocking, DMH^{LPB} blocking)<br>factor two: time<br>Uncorrected Fisher's LSD test	Virus: $F(1, 17) = 0.06235$, $P=0.8058$<br>Time: $F(2.683, 45.62) = 39.88$, $P<0.0001$<br>Interaction: $F(360, 6120) = 0.6782$, $P>0.9999$<br>Multiple comparisons: 19 min – 35 min, $P<0.0471$
3j	GFP: 10<br>DMH^{LPB} blocking: 10	RM two-way ANOVA<br>factor one: virus (GFP, DMH^{LPB} blocking)<br>factor two: time<br>Uncorrected Fisher's LSD test	Virus: $F(1, 18) = 34.35$, $P<0.0001$<br>Time: $F(2.993, 53.87) = 106.5$, $P<0.0001$<br>Interaction: $F(360, 6480) = 25.91$, $P<0.0001$<br>Multiple comparisons: 31 min – 300 min, $P<0.0008$
	POA^{LPB} blocking: 9<br>DMH^{LPB} blocking: 10	RM two-way ANOVA<br>factor one: virus (POA^{LPB} blocking, DMH^{LPB} blocking)<br>factor two: time<br>Uncorrected Fisher's LSD test	Virus: $F(1, 17) = 0.2659$, $P=0.6128$<br>Time: $F(2.421, 41.16) = 122.5$, $P<0.0001$<br>Interaction: $F(360, 6120) = 0.6928$, $P>0.9999$<br>Multiple comparisons: $P>0.2914$
	POA^{LPB} blocking: 9<br>Co-blocking: 6	RM two-way ANOVA<br>factor one: virus (POA^{LPB} blocking, Co-blocking)<br>factor two: time<br>Uncorrected Fisher's LSD test	Virus: $F(1, 13) = 2.316$, $P=0.1520$<br>Time: $F(1.727, 22.45) = 137.5$, $P<0.0001$<br>Interaction: $F(154, 2002) = 3.619$, $P<0.0001$<br>Multiple comparisons: 61 min – 94 min, $P<0.0482$
3k	GFP: 10<br>DMH^{LPB} blocking: 10	RM two-way ANOVA<br>factor one: virus (GFP, DMH^{LPB} blocking)<br>factor two: time<br>Uncorrected Fisher's LSD test	Virus: $F(1, 18) = 93.14$, $P<0.0001$<br>Time: $F(2.270, 40.86) = 158.3$, $P<0.0001$<br>Interaction: $F(277, 4986) = 46.53$, $P<0.0001$<br>Multiple comparisons: 31 min – 217 min, $P<0.0009$
	POA^{LPB} blocking: 9<br>DMH^{LPB} blocking: 10	RM two-way ANOVA<br>factor one: virus (POA^{LPB} blocking, DMH^{LPB} blocking)<br>factor two: time<br>Uncorrected Fisher's LSD test	Virus: $F(1, 17) = 0.02254$, $P=0.8824$<br>Time: $F(277, 4709) = 192.6$, $P<0.0001$<br>Interaction: $F(277, 4709) = 1.468$, $P<0.0001$<br>Multiple comparisons: 22 min – 37 min, $P<0.0429$
	POA^{LPB} blocking: 9<br>Co-blocking: 6	RM two-way ANOVA<br>factor one: virus (POA^{LPB} blocking, Co-blocking)<br>factor two: time<br>Uncorrected Fisher's LSD test	Virus: $F(1, 13) = 5.089$, $P=0.0419$<br>Time: $F(1.814, 23.58) = 166.2$, $P<0.0001$<br>Interaction: $F(120, 1560) = 4.740$, $P<0.0001$<br>Multiple comparisons: 36 min – 59 min, $P<0.0417$

(*table continued on the next page*)

Supplementary Table 2. Summary of statistical analyses

Figure	Sample size (n)	Statistical test	P values
4c	ChR2: 10 mice<br>GFP: 8 mice	RM two-way ANOVA<br>factor one: virus (ChR2, GFP)<br>factor two: time<br>Bonferroni's multiple comparisons test	Virus: *F (1, 16) = 11.26*, *P=0.0040*<br>Time: *F (90, 1440) = 7.309*, *P<0.0001*<br>Interaction: *F (90, 1440) = 12.99*, *P<0.0001*<br>Multiple comparisons: 8 min – 40 min, *P<0.0001*
4d	ChR2: 10 mice<br>GFP: 8 mice	RM two-way ANOVA<br>factor one: virus (ChR2, GFP)<br>factor two: time<br>Uncorrected Fisher's LSD test	Virus: *F (1, 16) = 30.92*, *P<0.0001*<br>Time: *F (90, 1440) = 4.328*, *P<0.0001*<br>Interaction: *F (90, 1440) = 1.737*, *P<0.0001*<br>multiple comparisons: Before-After: 0 min – 38 min, *P<0.0396*
4k	Non-blocking: 10 mice<br>DMH^{Vglut2} blocking: 5 mice<br>DMH^{Vgat} blocking: 5 mice<br>POA blocked: 5 mice	RM two-way ANOVA<br>factor one: treatment (Non-blocking, DMH^{Vglut2} blocking, DMH^{Vgat} blocking, POA blocking)<br>factor two: time<br>Bonferroni's multiple comparisons test	Treatment: *F (3, 21) = 9.102, P=0.0005*<br>Time: *F (90, 1890) = 11.60, P<0.0001*<br>Interaction: *F (270, 1890) = 10.46, P<0.0001*<br>Multiple comparisons:<br>Non-blocking vs. DMH^{Vglut2} blocking: 9 min – 45 min, *P<0.0001*<br>Non-blocking vs. DMH^{Vgat} blocking: 7 min – 43 min, *P<0.0001*<br>Non-blocking vs. POA blocking: *P>0.4970*
4l	Non-blocking: 10 mice<br>DMH^{Vglut2} blocking: 5 mice<br>DMH^{Vgat} blocking: 5 mice<br>GFP:5 mice	RM two-way ANOVA<br>factor one: treatment (Non-blocking, DMH^{Vglut2} blocking, DMH^{Vgat} blocking, GFP)<br>factor two: time<br>Bonferroni's multiple comparisons test	Treatment: *F (3, 21) = 9.566, P=0.0003*<br>Time: *F (1, 21) = 13.59, P=0.0014*<br>Interaction: *F (3, 21) = 7.305, P=0.0015*<br>Multiple comparisons:<br>b.s. vs. 10 min – 30 min:<br>Non-blocking: *P=0.0005*<br>GFP: *P>0.9999*<br>DMH^{Vglut2} blocking: *P=0.8948*<br>DMH^{Vgat} blocking: *P>0.9999*
4m	5 mice each	RM two-way ANOVA<br>factor one: treatment (Non-blocking, DMH^{Vglut2} blocking, DMH^{Vgat} blocking, GFP)<br>factor two: time<br>Bonferroni's multiple comparisons test	Treatment: *F (3, 16) = 23.36*, *P<0.0001*<br>Time: *F (9, 144) = 1.652*, *P=0.1060*<br>Interaction: *F (27, 144) = 8.832*, *P<0.0001*<br>Multiple comparisons:<br>GFP vs. Non-blocking: 8 min – 60 min, *P<0.0002*<br>GFP vs. DMH^{Vgat} blocking: 8 min – 30 min, *P<0.016*<br>GFP vs. DMH^{Vglut2} blocking: *P>0.3106*
4n	4 mice each	RM two-way ANOVA<br>factor one: treatment (Non-blocking, DMH^{Vglut2} blocking, DMH^{Vgat} blocking)<br>factor two: time<br>Bonferroni's multiple comparisons test	Treatment: *F (3, 12) = 26.85*, *P<0.0001*<br>Time: *F (12, 144) = 9.665*, *P<0.0001*<br>Interaction: *F (36, 144) = 9.762*, *P<0.0001*<br>Multiple comparisons:<br>Non-blocking vs. DMH^{Vglut2} blocking: 40 s – 60 s, *P<0.0001*<br>Non-blocking vs. DMH^{Vgat} blocking: 40 s – 60 s, *P<0.0001*<br>Non-blocking vs. GFP: 40 – 60 s, *P<0.0001*
4o	5 mice each	RM two-way ANOVA<br>factor one: treatment (Non-blocking, DMH^{Vglut2} blocking, DMH^{Vgat} blocking,GFP)<br>factor two: time<br>Bonferroni's multiple comparisons test	Treatment: *F (3, 16) = 3.472*, *P=0.0410*<br>Time: *F (90, 1440) = 1.897*, *P<0.0001*<br>Interaction: *F (270, 1440) = 2.218*, *P<0.0001*<br>Multiple comparisons:<br>Non-blocking vs. GFP: 1 – 14 min, *P*<0.01<br>Non-blocking vs. DMH^{Vglut2} blocking: 1 min – 16 min, *P*<0.01<br>Non-blocking vs. DMH^{Vgat} blocking: 1 min – 27 min, *P*<0.01
4q	ChR2: 6mice	RM one-way ANOVA<br>factor one: treatment (ChR2: 24ºC, ChR2: 6ºC, ChR2: 30ºC,)<br>Bonferroni's multiple comparisons test	Treatment: *F (2, 15) = 1.890*, *P=0.1854*<br>Multiple comparisons:<br>30 ºC vs. 24 ºC: *P=0.7829*<br>24 ºC vs. 6 ºC: *P=0.7706*
4r	ChR2: 6mice	RM one-way ANOVA<br>factor one: treatment (ChR2: 24ºC, ChR2: 6ºC, ChR2: 30ºC,)<br>Bonferroni's multiple comparisons test	Treatment: *F (2, 10) = 14.57*, *P=0.0011*<br>Multiple comparisons:<br>30 ºC vs. 6 ºC: *P=0.0012*<br>24 ºC vs. 6 ºC: *P=0.0027*
5c	GFP: 9 mice<br>ChR2: 9 mice	Two-tailed unpaired t test	t=3.205, df=16, *P=0.0055*
5g	ChR2: 9 mice<br>GFP: 5 mice	RM two-way ANOVA<br>factor one: virus (ChR2, GFP)<br>factor two: time<br>Uncorrected Fisher's LSD test	Virus: *F (1, 12) = 1.604*, *P=0.2294*<br>Time: *F (33, 396) = 25.36*, *P<0.0001*<br>Interaction: *F (33, 396) = 1.439*, *P=0.0590*<br>Multiple comparisons: 10 -16 day, *P<0.0261*
5h	ChR2: 9 mice<br>GFP: 5 mice	RM two-way ANOVA<br>factor one: treatment (GFP, ChR2)<br>factor two: time<br>Bonferroni's multiple comparisons test	Treatment: *F (1, 168) = 32.50*, *P<0.0001*<br>Time: *F (13, 168) = 141.0*, *P<0.0001*<br>Interaction: *F (13, 168) = 0.6262*, *P=0.8299*<br>Multiple comparisons:<br>*P>0.1307*
5i	GFP: 6 mice<br>ChR2: 6 mice	Two-tailed unpaired t test	t=3.348, df=10, *P=0.0074*
5j	GFP: 6 mice<br>ChR2: 6 mice	Two-tailed unpaired t test	t=2.235, df=10, *P=0.0494*
5k	ChR2: 6 mice<br>GFP: 6 mice	Ordinary two-way ANOVA<br>factor one: virus (ChR2, GFP)<br>factor two: tissues (fat, lean)<br>Bonferroni's multiple comparisons test	Virus: *F (1, 20) = 5.225*, *P=0.0333*<br>Tissues: *F (1, 20) = 130.0*, *P<0.0001*<br>Interaction: *F (1, 20) = 13.16*, *P=0.0017*<br>Multiple comparisons:<br>Fat: ChR2 vs. GFP: *P=0.0009*<br>Lean: ChR2 vs. GFP: *P=0.7085*
5l	ChR2: 9 mice<br>GFP: 5 mice	Ordinary two-way ANOVA<br>factor one: virus (ChR2, GFP)<br>factor two: time (-200-0, 0-400)<br>Šídák's multiple comparisons test	Virus: *F (1, 12) = 4.963*, *P=0.0458*<br>Time: *F (1, 12) = 354.4*, *P<0.0001*<br>Interaction: *F (1, 12) = 4.062*, *P=0.0668*<br>Multiple comparisons:<br>-200 min - 0 min: *P*=0.5522<br>0 min - 400 min: *P*=0.0149

(*table continued on the next page*)

Supplementary Table 2. Summary of statistical analyses

Figure	Sample size (n)	Statistical test	P values
7c	ChR2: 8 mice<br>GFP: 7 mice	RM two-way ANOVA<br>factor one: virus (ChR2, GFP)<br>factor two: time<br>Bonferroni's multiple comparisons test	Virus: $F(1, 13) = 8.669$, $P=0.0114$<br>Time: $F(90, 1170) = 10.24$, $P<0.0001$<br>Interaction: $F(90, 1170) = 8.463$, $P<0.0001$<br>Multiple comparisons:<br>14 min – 49 min: $P<0.0061$
7d	ChR2: 8 mice<br>GFP:7 mice	RM two-way ANOVA<br>factor one: virus (ChR2, GFP)<br>factor two: time<br>Bonferroni's multiple comparisons test	Virus: $F(1, 13) = 0.1.853$, $P=0.1965$<br>Time: $F(90, 1170) = 1.020$, $P=0.4314$<br>Interaction: $F(90, 1170) = 0.8949$, $P=0.7459$<br>Multiple comparisons: $P>0.9999$
7e	ChR2: 6 mice<br>GFP: 7 mice	RM two-way ANOVA<br>factor one: virus (ChR2, GFP)<br>factor two: time<br>Bonferroni's multiple comparisons test	Virus: $F(1, 11) = 31.63$, $P=0.0002$<br>Time: $F(18, 198) = 7.932$, $P<0.0001$<br>Interaction: $F(18, 198) = 7.532$, $P<0.0001$<br>Multiple comparisons:<br>5 min – 40 min: $P<0.0037$
7f	Sham: 6 mice<br>Denervation: 6 mice	RM two-way ANOVA<br>factor one: treatment (Sham, Denervation)<br>factor two: time<br>Bonferroni's multiple comparisons test	Treatment: $F(1, 10) = 3.611$, $P=0.0866$<br>Time: $F(89, 890) = 12.18$, $P<0.0001$<br>Interaction: $F(89, 890) = 5.664$, $P<0.0001$<br>Multiple comparisons:<br>22 min – 34 min: $P<0.0390$
7g	SST: 4 mice<br>Vglut2: 4 mice	RM two-way ANOVA<br>factor one: mice (SST, Vglut2)<br>factor two: time<br>Bonferroni's multiple comparisons test	Mice: $F(1, 6) = 259.6$, $P<0.0001$<br>Time: $F(12, 72) = 9.856$, $P<0.0001$<br>Interaction: $F(12, 72) = 8.922$, $P<0.0001$<br>Multiple comparisons:<br>40 s – 60 s, $P<0.0001$
7h	SST: 8 mice<br>Vglut2: 5 mice	RM two-way ANOVA<br>factor one: mice (SST, Vglut2)<br>factor two: time<br>Bonferroni's multiple comparisons test	Mice: $F(2, 15) = 1.564$, $P=0.2416$<br>Time: $F(89, 1335) = 1.907$, $P<0.0001$<br>Interaction: $F(178, 1335) = 2.628$, $P<0.0001$<br>Multiple comparisons:<br>0 min – 16 min, $P<0.0012$
7k	ChR2: 6 mice<br>ChR2 + TeNT:6 mice	RM two-way ANOVA<br>factor one: virus (ChR2, ChR2 + TeNT)<br>factor two: time<br>Bonferroni's multiple comparisons test	Virus: $F(1, 10) = 0.04145$, $P=0.8428$<br>Time: $F(2.483, 24.83) = 0.04145$, $P<0.0001$<br>Interaction: $F(18, 180) = 0.8825$, $P=6003$<br>Multiple comparisons: $P>0.9878$
7l	ChR2: 6 mice<br>ChR2 + TeNT:6 mice	RM two-way ANOVA<br>factor one: virus (ChR2, ChR2 + TeNT)<br>factor two: time<br>Bonferroni's multiple comparisons test	Virus: $F(1, 10) = 0.4483$, $P=0.5183$<br>Time: $F(4.334, 43.34) = 21.94$, $P<0.0001$<br>Interaction: $F(18, 180) = 3.162$, $P<0.0001$<br>Multiple comparisons: $P>0.9999$
7n	taCasp3: 8 mice<br>mCherry: 8 mice	RM two-way ANOVA<br>factor one: virus (taCasp3, mCherry)<br>factor two: time<br>Bonferroni's multiple comparisons test	Virus: $F(1, 14) = 0.01322$, $P=0.7216$<br>Time: $F(60, 840) = 35.12$, $P<0.0001$<br>Interaction: $F(60, 840) = 0.5366$, $P=0.9985$<br>Multiple comparisons: $P>0.9999$
7o	taCasp3: 8 mice<br>mCherry: 8 mice	RM two-way ANOVA<br>factor one: virus (taCasp3, mCherry)<br>factor two: time<br>Bonferroni's multiple comparisons test	Virus: $F(1, 14) = 12.95$, $P=0.0029$<br>Time: $F(60, 455) = 47.2$, $P<0.0001$<br>Interaction: $F(60, 775) = 12.17$, $P<0.0001$<br>Multiple comparisons: 180 min – 300 min, $P<0.0014$
7r	Non-blocking: 8 mice<br>$DMH^{Lep\&SST-TeNT}$: 7 mice<br>$DMH^{ChAT\&SST-TeNT}$: 7 mice	RM two-way ANOVA<br>factor one: treatment (Non-blocking, $DMH^{Lep\&SST-TeNT}$, $DMH^{ChAT\&SST-TeNT}$)<br>factor two: time<br>Bonferroni's multiple comparisons test	Virus: $F(1, 13) = 11.18$, $P=0.0053$<br>Time: $F(90, 1170) = 13.96$, $P<0.0001$<br>Interaction: $F(90, 1170) = 4.768$, $P<0.0001$<br>Multiple comparisons:<br>Non-blocking vs. $DMH^{Lep\&SST-TeNT}$, 11 min – 51 min, $P<0.0381$

(*table continued on the next page*)

Supplementary Table 2. Summary of statistical analyses

Figure	Sample size (n)	Statistical test	P values
S5d	GFP: 7 mice<br>DMH^{LPB} blocking: 7 mice<br>POA^{LPB} blocking: 7 mice	Ordinary one-way ANOVA<br>factor one: virus (GFP, DMH^{LPB} blocking, POA^{LPB} blocking)<br>Tukey's multiple comparisons test	Treatment: *F (2, 3) = 0.4269*, *P=0.6868*<br>Multiple comparisons:<br>DMH^{LPB} blocking vs. POA^{LPB} blocking: *P=0.6663*<br>GFP vs. POA^{LPB} blocking: *P=0.9211*<br>DMH^{LPB} blocking vs. GFP: *P=0.8634*
S5e	POA^{LPB}-blocking: 7 mice<br>DMH^{LPB}-blocking: 7 mice<br>GFP control: 7 mice	Ordinary one-way ANOVA<br>factor: treatment (POA^{LPB}-blocking, DMH^{LPB}-blocking, GFP control)<br>Tukey's multiple comparisons test	Dark:<br>Treatment: F (2, 18) = 12.89, P=0.0003<br>Multiple comparisons:<br>POA^{LPB} blocking vs. DMH^{LPB} blocking, P=0.0008<br>POA^{LPB}blocking vs. GFP, P=0.0012<br>DMH^{LPB} blocking vs. GFP, P=0.9845<br>Light:<br>Treatment: F (2, 18) = 11.33,P=0.0007<br>Multiple comparisons:<br>POA^{LPB} blocking vs. DMH^{LPB} blocking, P=0.0385<br>POA^{LPB} blocking vs. GFP, P=0.0005<br>DMH^{LPB} blocking vs. GFP, P=0.1258
S5f	POA^{LPB}-blocking: 7 mice<br>DMH^{LPB}-blocking: 7 mice<br>GFP control: 7 mice	Ordinary one-way ANOVA<br>factor: treatment (POA^{LPB}-blocking, DMH^{LPB}-blocking, GFP control)<br>Tukey's multiple comparisons test	Dark:<br>Treatment: F (2, 18) = 3.286, P=0.0607<br>Multiple comparisons:<br>POA^{LPB} blocking vs. DMH^{LPB} blocking, P=0.1670<br>POA^{LPB}blocking vs. GFP, P=0.8535<br>DMH^{LPB} blocking vs. GFP, P=0.0623<br>Light:<br>Treatment: F (2, 18) = 7.078,P=0.0054<br>Multiple comparisons:<br>POA^{LPB} blocking vs. DMH^{LPB} blocking, P=0.0678<br>POA^{LPB} blocking vs. GFP, P=0.0043<br>DMH^{LPB} blocking vs. GFP, P=0.4060
S7g	GFP: 7 mice<br>TeNT:9 mice	RM two-way ANOVA<br>factor one: virus (GFP, TeNT)<br>factor two: time<br>Šídák's multiple comparisons test	Virus: *F (1, 14) = 30.61*<br>Time: *F (2.366, 32.19) = 23.09*,<br>*Geisser-Greenhouse's epsilon, 0.04929*<br>Interaction: *F (48, 653) = 11.95*<br>Multiple comparisons:<br>90 min – 150 min, *P*<0.0152
S7j	GFP: 5 mice<br>TeNT:7 mice	RM two-way ANOVA<br>factor one: virus (GFP, TeNT)<br>factor two: time<br>Bonferroni's multiple comparisons test	Virus: *F (1, 10) = 17.18*, *P=0.0020*<br>Time: *F (31, 310) = 10.98*, *P<0.0001*<br>Interaction: *F (31, 310) = 4.533*, *P<0.0001*<br>Multiple comparisons:<br>70 min – 140 min, *P*<0.0288
S7m	GFP: 4 mice<br>TeNT:7 mice	RM two-way ANOVA<br>factor one: virus (GFP, TeNT)<br>factor two: time<br>Bonferroni's multiple comparisons test	Virus: *F (1, 9) = 5.940*, *P=0.0375*<br>Time: *F (48, 432) = 28.06*, *P<0.0001*<br>Interaction: *F (48, 432) = 7.574*, *P<0.0001*<br>Multiple comparisons:<br>90 min – 180 min, P<0.0254
S9d	mCherry: 6 mice<br>ChR2: 6 mice	RM two-way ANOVA<br>factor one: virus (mCherry, ChR2)<br>factor two: time<br>Uncorrected Fisher's LSD test	Time: F (2.154, 21.54) = 5.879, P=0.0081<br>Virus: F (1, 10) = 5.952, P=0.0349<br>Interaction: F (90, 900) = 4.427, P<0.0001<br>Multiple comparisons:<br>13 min – 42 min, *P*<0.0455
S9g	Saline: 7 mice<br>CNO: 7 mice	RM two-way ANOVA<br>factor one: treatment (Saline, CNO)<br>factor two: time<br>Šídák's multiple comparisons test	Time: F (3.850, 46.20) = 13.31, P<0.0001<br>Virus: F (1, 12) = 14.28, P=0.0026<br>Interaction: F (21, 252) = 3.975, P<0.0001<br>Multiple comparisons:<br>20 min – 40 min, P<0.0204<br>60 min – 90 min, P<0.0199
S9i	Saline: 12 mice<br>CNO: 12 mice	RM two-way ANOVA<br>factor one: treatment (Saline, CNO)<br>factor two: time<br>Šídák's multiple comparisons test	Time: F (5.349, 117.7) = 4.384, P=0.0008<br>Virus: F (1, 22) = 28.07, P<0.0001<br>Interaction: F (21, 462) = 3.374, P<0.0001<br>Multiple comparisons:<br>20 min – 130 min, P<0.0313<br>150 min, P=0.0076<br>170 min-180 min, P<0.0018

(*table continued on the next page*)

Supplementary Table 2. Summary of statistical analyses

Figure	Sample size (n)	Statistical test	P values
S10b	GFP: 6 mice<br>ChR2: 9 mice	RM two-way ANOVA<br>factor one: virus (GFP, ChR2)<br>factor two: time<br>Uncorrected Fisher's LSD test	Time: F (3.876, 50.39) = 11.51, P<0.0001<br>Virus: F (1, 13) = 1.662, P=0.2198<br>Interaction: F (90, 1170) = 7.278, P<0.0001<br>Multiple comparisons:<br>4 min – 35 min, *P*<0.0418
S10f	mCherry: 6 mice<br>TeNT: 6 mice	Two-tailed unpaired t test	t=1.293, df=10,<br>*P=0.2251*
	mCherry: 6 mice<br>TeNT: 6 mice	Two-tailed unpaired t test	t=1.677, df=10,<br>*P=0.1245*
S10g	mCherry: 7 mice<br>TeNT:6 mice	RM two-way ANOVA<br>factor one: virus (mCherry, TeNT)<br>factor two: time<br>Uncorrected Fisher's LSD test	Virus: *F (1, 11) = 7.392*, *P=0.0200*<br>Time: *F (90.00, 990.0) = 2.188*, *P<0.0001*<br>Interaction: *F (90, 990) = 8.050*, *P<0.0001*<br>Multiple comparisons:<br>7 min – 42 min, *P*<0.0415
S10h	mCherry: 7 mice<br>TeNT: 6 mice	RM two-way ANOVA<br>factor one: virus (mCherry, TeNT)<br>factor two: time<br>Uncorrected Fisher's LSD test	Virus: *F (1, 11) = 4.405*, *P=0.0597*<br>Time: *F* (90, 990) = 3.534, *P<0.0001*<br>Interaction: *F (90, 990) = 2.707*, *P<0.0001*<br>Multiple comparisons:<br>1 min – 3 min, *P*<0.0448<br>6 min – 19 min, *P*<0.0457<br>21 min – 24 min, *P*<0.0404<br>30 min, P=0.0406<br>33 min – 34 min, *P*<0.0335
S10i	mCherry: 5 mice<br>TeNT: 4 mice	RM two-way ANOVA<br>factor one: virus (mCherry, TeNT)<br>factor two: time<br>Šídák's multiple comparisons test	Virus: *F (1, 7) = 5.880*, *P=0.0458*<br>Time: *F* (2.537, 17.76) = 4.565, *P=0.0190*<br>Interaction: *F (11, 77) = 2.039*, *P=0.0356*<br>Multiple comparisons:<br>10 min, *P*=0.0217
S10j	mCherry: 5 mice<br>TeNT: 4 mice	RM two-way ANOVA<br>factor one: virus (mCherry, TeNT)<br>factor two: time<br>Šídák's multiple comparisons test	Virus: *F (1, 7) = 1.922*, *P=0.2082*<br>Time: *F* (6, 42) = 3.137, *P=0.0124*<br>Interaction: *F (6, 42) = 3.162*, *P=0.0119*<br>Multiple comparisons:<br>80 min, *P*=0.0344
S10m	GFP: 6 mice<br>TeNT: 5 mice	RM two-way ANOVA<br>factor one: virus (GFP, TeNT)<br>factor two: time<br>Uncorrected Fisher's LSD test	Virus: *F (1, 9) = 3.135*, *P=0.1104*<br>Time: *F (1, 9) = 24.71*, *P=0.0008*<br>Interaction: *F (1, 9) = 3.036*, *P=0.1154*<br>Multiple comparisons:<br>Light, *P*=0.5038<br>Dark, *P*=0.0275
	GFP: 6 mice<br>TeNT: 5 mice	RM two-way ANOVA<br>factor one: virus (GFP, TeNT)<br>factor two: time<br>Uncorrected Fisher's LSD test	Virus: *F (1, 9) = 3.809*, *P=0.0827*<br>Time: *F* (1, 9) = 13.09, *P=0.0056*<br>Interaction: *F (1, 9) = 1.791*, *P=0.2137*<br>Multiple comparisons:<br>Light, *P*=0.6648<br>Dark, *P*=0.0318
S10n	GFP: 6 mice<br>TeNT: 6 mice	RM two-way ANOVA<br>factor one: virus (GFP, TeNT)<br>factor two: time<br>Šídák's multiple comparisons test	Virus: *F (1, 10) = 34.35*, *P=0.0002*<br>Time: *F (3.926, 39.26) = 6.699*, *P=0.0004*<br>Interaction: *F (91, 910) = 6.330*, *P<0.0001*<br>Multiple comparisons:<br>11 min-18 min, P<0.0463<br>37 min-38 min, P<0.0418<br>41 min, P=0.0476
S10o	GFP: 6 mice<br>TeNT: 6 mice	RM two-way ANOVA<br>factor one: virus (GFP, TeNT)<br>factor two: time<br>Šídák's multiple comparisons test	Virus: *F (1, 10) = 9.150*, *P=0.0128*<br>Time: *F (1,10) = 19.65*, *P=0.0013*<br>Interaction: *F (1, 10) = 0.3412*, *P=0.5721*<br>Multiple comparisons:<br>-30-0 min VS. 0-30 min: TeNT, *P=0.0106*<br>GFP, P=0.0426<br>TeNT VS. GFP: -30-0 min, *P=0.0229*<br>0-30 min, *P=0.0119*
