# Supplementary material for: A parabrachial-hypothalamic parallel circuit governs cold defense in mice
Source: Nat Commun. 2023 Aug 15;14:4924. doi: 10.1038/s41467-023-40504-6 (PMC10427655; doi:10.1038/s41467-023-40504-6)
Supplement: Supplementary file 3 — Reporting Summary [file 41467_2023_40504_MOESM3_ESM.pdf]

nature portfolio

Corresponding author(s): Wei. L. Shen

Last updated by author(s): Jul 9, 2023

Reporting Summary

Nature Portfolio wishes to improve the reproducibility of the work that we publish. This form provides structure for consistency and transparency in reporting. For further information on Nature Portfolio policies, see our Editorial Policies and the Editorial Policy Checklist.

Statistics

For all statistical analyses, confirm that the following items are present in the figure legend, table legend, main text, or Methods section.

n/a	Confirmed	
☐	☒	The exact sample size (*n*) for each experimental group/condition, given as a discrete number and unit of measurement
☐	☒	A statement on whether measurements were taken from distinct samples or whether the same sample was measured repeatedly
☐	☒	The statistical test(s) used AND whether they are one- or two-sided<br>*Only common tests should be described solely by name; describe more complex techniques in the Methods section.*
☒	☐	A description of all covariates tested
☒	☐	A description of any assumptions or corrections, such as tests of normality and adjustment for multiple comparisons
☐	☒	A full description of the statistical parameters including central tendency (e.g. means) or other basic estimates (e.g. regression coefficient) AND variation (e.g. standard deviation) or associated estimates of uncertainty (e.g. confidence intervals)
☐	☒	For null hypothesis testing, the test statistic (e.g. *F*, *t*, *r*) with confidence intervals, effect sizes, degrees of freedom and *P* value noted<br>*Give P values as exact values whenever suitable.*
☒	☐	For Bayesian analysis, information on the choice of priors and Markov chain Monte Carlo settings
☒	☐	For hierarchical and complex designs, identification of the appropriate level for tests and full reporting of outcomes
☒	☐	Estimates of effect sizes (e.g. Cohen's *d*, Pearson's *r*), indicating how they were calculated

Our web collection on statistics for biologists contains articles on many of the points above.

Software and code

Policy information about availability of computer code

Data collection	The following softwares were used to collect data: oliVIA 3.2; Nikon A1R 3.2; Axon MultiClamp 700B; Fscope Fiber Photometry System 1.0; SPIKES2 9.0; Digbehv video-recording software 2.0; VITALVIEW 5.0; Columbus Clams 5.4; FLIR Tools software 5.6
Data analysis	The following softwares were used to analyze data: Digbehv video-recording software 2.0; FLIR Tools software 5.6; Image J 1.53; Spikes2 9.0; Excel 2019; Graphpad Prism 8; Clampex 10 , Matlab 2021b

For manuscripts utilizing custom algorithms or software that are central to the research but not yet described in published literature, software must be made available to editors and reviewers. We strongly encourage code deposition in a community repository (e.g. GitHub). See the Nature Portfolio guidelines for submitting code & software for further information.

Data

Policy information about availability of data

All manuscripts must include a data availability statement. This statement should provide the following information, where applicable:

- Accession codes, unique identifiers, or web links for publicly available datasets
- A description of any restrictions on data availability
- For clinical datasets or third party data, please ensure that the statement adheres to our policy

Source data are provided with this paper and has been deposited in Figshare repository under accession code https://doi.org/10.6084/m9.figshare.23652297

Human research participants

Policy information about studies involving human research participants and Sex and Gender in Research.

Reporting on sex and gender	N/A
Population characteristics	N/A
Recruitment	N/A
Ethics oversight	N/A

Note that full information on the approval of the study protocol must also be provided in the manuscript.

Field-specific reporting

Please select the one below that is the best fit for your research. If you are not sure, read the appropriate sections before making your selection.

☒ Life sciences ☐ Behavioural & social sciences ☐ Ecological, evolutionary & environmental sciences

For a reference copy of the document with all sections, see nature.com/documents/nr-reporting-summary-flat.pdf

Life sciences study design

All studies must disclose on these points even when the disclosure is negative.

Sample size	The minimal sample size was pre-determined by the nature of experiments. No statistical methods were used to pre-determine sample sizes but our sample sizes are similar to those reported in previous publications (10.1038/s41593-018-0249-3, 10.1038/s41586-020-2163-6). For most of physiological readouts (body temperature, physical activity, brown fat temperature, heart rate and electrogram etc.), 5-20 different mice per group were included. For histology studies, the same experiment was repeated for three times. For fiber photometry recording, 6-10 mice and 2-5 trials for each mice were included.For electrophysiological studies, at least 13 different neurons from 3 different mice were included.
Data exclusions	Data from mice (often 0-20%) with few or no viral expression were removed.
Replication	Each experiment was successfully replicated in least three independent biological individuals
Randomization	Allocation of organisms and brain slices into experimental groups was done at random. All conditions were randomly counterbalanced across organisms and brain slices
Blinding	The experimenter was blinded to genotype/experimental group before and during all experiments.

Reporting for specific materials, systems and methods

We require information from authors about some types of materials, experimental systems and methods used in many studies. Here, indicate whether each material, system or method listed is relevant to your study. If you are not sure if a list item applies to your research, read the appropriate section before selecting a response.

Materials & experimental systems

n/a	Involved in the study	
☐	☒	Antibodies
☒	☐	Eukaryotic cell lines
☒	☐	Palaeontology and archaeology
☐	☒	Animals and other organisms
☒	☐	Clinical data
☒	☐	Dual use research of concern

Methods

n/a	Involved in the study	
☒	☐	ChIP-seq
☒	☐	Flow cytometry
☒	☐	MRI-based neuroimaging

Antibodies

Antibodies used	1.Chicken anti-GFP : Abcam, Cat# ab13970 RRID: AB_300798;<br>2.Guinea pig anti-cFos: Synaptic systems Cat# 226004 RRID: AB_2619946;

3.Rabbit anti-cFos : Synaptic systems Cat# 226003 RRID: AB_2231974;
4.Rat anti-RFP: Chromotek Cat# 5F8 RRID: AB_2336064;
5.Rabbit anti-Somatostatin: ImmunoStar Cat# 20067 RRID: AB_572264;
6.Rabbit anti-Glutamate : Sigma Cat# G6642 RRID: AB_259946;
7.Rabbit anti-GABA : Sigma Cat# A2052 RRID: AB_477652;
8.DyLight 488 conjugated goat anti-chicken: Invitrogen Cat# SA5-10070 RRID: AB_2556650;
9. Alexa Fluor 594 conjugated goat anti-rat IgG: Invitrogen Cat# A-11007 RRID: AB_141374;
10.Alexa Fluor 594 conjugated goat anti-Guinea pig IgG: Invitrogen Cat#A-11076 RRID: AB_ 141930;
11.Alexa Fluor 594 conjugated goat anti-rabbit IgG: Jackson Cat# 111-585-144 RRID: AB_2307325;
12.Alexa Fluor 488 conjugated goat anti-rabbit IgG: Jackson Cat# 111-545-003 RRID: AB_2338046;
13.Alexa Fluor 647 conjugated goat anti-rabbit: Invitrogen Cat# A21244 RRID: AB_141663;
all above were used for immunostainning.
anti-EGFP antibody from Monoclonal Antibody Core Facility at Memorial Sloan-Kettering cancer center an with equal mixture of clones 19C8 and 19F7 was applied for immunoprecipitation.

Validation

Validation was provided through the manufacturer's data sheet (see above for manufacturer and product code information) and/or the published use and validation of the relevant antibodies by our and other research groups. Here, we list some selected references and web links of these products information.
1.Chicken anti-GFP Cat# ab13970 has been referenced in 3181 publications.
(1)Berg EM et al. Brainstem circuits encoding start, speed, and duration of swimming in adult zebrafish. Neuron 111:372-386.e4 (2023).
(2) https://www.abcam.com/products/primary-antibodies/gfp-antibody-ab13970.html
2.Guinea pig anti-cFos Synaptic systems Cat# 226004 has been used and validated in other studies.
(1) van Heukelum, S., et al. (2021). "A central role for anterior cingulate cortex in the control of pathological aggression." Curr Biol 31(11): 2321-2333 e2325.
3.Rabbit anti-cFos Synaptic systems Cat# 226003 has been used and validated in other studies.
(1) Noye Tuplin, E. W., et al. (2018). "Comparison of the Time-Dependent Changes in Immediate Early Gene Labeling and Spine Density
(2) Following Abstinence From Contingent or Non-contingent Chocolate Pellet Delivery." Front Behav Neurosci 12: 144.
Dumrongprechachan, V., et al. (2021). "Cell-type and subcellular compartment-specific APEX2 proximity labeling reveals activity-dependent nuclear proteome dynamics in the striatum." Nat Commun 12(1): 4855.
4.Rat anti-RFP: Chromotek Cat# 5F8 has been referenced in 558 publications,
(1) Zhang, Y., et al. (2023). "Fast and sensitive GCaMP calcium indicators for imaging neural populations." Nature 615(7954): 884-891.
(2) https://www.ptglab.com/products/RFP-antibody-5F8.htm#publications
5.Rabbit anti-Somatostatin: ImmunoStar Cat# 20067 has been referenced in 314 publications,
(1) https://www.immunostar.com/product/somatostatin-antibody/
6.Rabbit anti-Glutamate : Sigma Cat# G6642 has been referenced in 36 publications
(1) Cell-type-specific imaging of neurotransmission reveals a disrupted excitatory-inhibitory cortical network in isoflurane anaesthesia.
Juan Guo et al. EBioMedicine, 65, 103272-103272 (2021-03-11)
(2) https://www.sigmaaldrich.cn/CN/zh/product/sigma/g6642
7.Rabbit anti-GABA : Sigma Cat# A2052 has been referenced in 562 publications
(1) Neuronal gamma-aminobutyric acid (GABA) type A receptors undergo cognate ligand chaperoning in the endoplasmic reticulum by endogenous GABA.Ping Wang et al. Frontiers in cellular neuroscience, 9, 188-188 (2015-06-05)
(2) https://www.sigmaaldrich.cn/CN/zh/product/sigma/a2052
8.anti-EGFP antibody from Monoclonal Antibody Core Facility at Memorial Sloan-Kettering cancer center an with equal mixture of clones 19C8 and 19F7 has been used and validated by other group.
(1) Tan, C. L., et al. (2016). "Warm-Sensitive Neurons that Control Body Temperature." Cell 167(1): 47-+.

Animals and other research organisms

Policy information about studies involving animals; ARRIVE guidelines recommended for reporting animal research, and Sex and Gender in Research

Laboratory animals	species: mus musculus. strains: C57BL/6J, Vgat-IRES-Cre, Vgat-T2A-FlpO, Vglut2-IRES-Cre, SST-IRES-Cre, LepR-Cre, AI14, ChAT-Cre (all from Jackson Laboratory); ages:8-16 weeks. Mice were housed under controlled temperature (22–25 °C) and humidity (50-70%) unless specified in a 12-hour reverse light/dark cycle (light time, 9 pm to 9 am) with food and water ad libitum. Mice were fed either a high fat diet (HFD, Research Diets, #D12492) or regular chow food (SLAC, #M03).
Wild animals	No wild animals were used in the study.
Reporting on sex	Male
Field-collected samples	No field collected samples were used in the study.
Ethics oversight	Animal care and use conformed to institutional guidelines of ShanghaiTech University, Shanghai Biomodel Organism Co., and governmental regulations.

Note that full information on the approval of the study protocol must also be provided in the manuscript.
